# Interpretable Deep Learning Model Reveals Subsequences of Various Functions for Long Non-Coding RNA Identification

**DOI:** 10.3389/fgene.2022.876721

**Published:** 2022-05-24

**Authors:** Rattaphon Lin, Duangdao Wichadakul

**Affiliations:** ^1^ Department of Computer Engineering, Faculty of Engineering, Chulalongkorn University, Pathumwan, Thailand; ^2^ Center of Excellence in Systems Biology, Faculty of Medicine, Chulalongkorn University, Pathumwan, Thailand

**Keywords:** long non-coding RNA (lncRNA), one-dimensional convolutional neural network (1D CNN), deep learning, explainable artificial intelligence (XAI), SHAP (SHapley additive exPlanations)

## Abstract

Long non-coding RNAs (lncRNAs) play crucial roles in many biological processes and are implicated in several diseases. With the next-generation sequencing technologies, substantial unannotated transcripts have been discovered. Classifying unannotated transcripts using biological experiments are more time-consuming and expensive than computational approaches. Several tools are available for identifying long non-coding RNAs. These tools, however, did not explain the features in their tools that contributed to the prediction results. Here, we present Xlnc1DCNN, a tool for distinguishing long non-coding RNAs (lncRNAs) from protein-coding transcripts (PCTs) using a one-dimensional convolutional neural network with prediction explanations. The evaluation results of the human test set showed that Xlnc1DCNN outperformed other state-of-the-art tools in terms of accuracy and F1-score. The explanation results revealed that lncRNA transcripts were mainly identified as sequences with no conserved regions, short patterns with unknown functions, or only regions of transmembrane helices while protein-coding transcripts were mostly classified by conserved protein domains or families. The explanation results also conveyed the probably inconsistent annotations among the public databases, lncRNA transcripts which contain protein domains, protein families, or intrinsically disordered regions (IDRs). Xlnc1DCNN is freely available at https://github.com/cucpbioinfo/Xlnc1DCNN.

## 1 Introduction

Long non-coding RNAs (lncRNAs) are RNAs that are not translated into proteins and are longer than 200 nucleotides. lncRNAs play important roles in many critical biological processes, including gene expression, gene regulation, gene silencing, chromatin remodeling, acting as molecular scaffolds, etc. (Rinn and Chang, 2012; Marchese et al., 2017; Statello et al., 2021), and have been implicated in human diseases such as cancers and diabetes (Morán et al., 2012; Fang and Fullwood, 2016; Chan and Tay, 2018; Jin et al., 2020). The enhancements of next-generation sequencing technology, i.e., RNA sequencing (RNA-Seq) (Wang et al., 2009; Stark et al., 2019) have led to numerous discoveries of unannotated transcripts. However, classifying the innumerable number of unclassified sequences using experimental approaches is time-consuming and expensive. In contrast, computational approaches are faster and more convenient.

Most of the existing computational approaches for classifying lncRNA and protein-coding transcripts used feature extraction methods to obtain training features, e.g., the upgraded version of Coding Potential Calculator (CPC2) (Kang et al., 2017), CNIT (Guo et al., 2019), PLEK (Li et al., 2014), CPAT (Wang et al., 2013), FEELnc (Wucher et al., 2017), RNAsamba (Camargo et al., 2020), LncADeep (Yang et al., 2018), and lncRNA_Mdeep (Fan et al., 2020). Most of them used similar features such as the Fickett and hexamer scores, the ORF length, and then topped up with additional sequence and structural features. Moreover, none of them explained how the features contributed to the model prediction results.

Deep learning algorithms have become very popular, especially for a dataset with a large number of data points and data dimensions as the features will be learned by the algorithms themselves during the training. Many convolutional neural networks (CNNs), the 2D-CNNs, have been widely used for image classification and segmentation applications (Yamashita et al., 2018) because of their great capability for extracting features from input data. Recently, many applications such as speech recognition and ECG monitoring (Kiranyaz et al., 2021) started using 1D-CNN instead of the traditional machine learning approaches. The applications for detecting irregular heartbeats (Acharya et al., 2017; Li et al., 2019; Hsieh et al., 2020) have shown that using only a simple 1D-CNN could achieve high prediction accuracy without explicitly addressing and extracting features as inputs for the models.

While most complex black-box models (e.g., boosting tree algorithms, ensemble models, deep neural networks) typically provide better learning performance, they usually are uninterpretable. To understand how a complex model learns to differentiate things, explainable artificial intelligence (XAI) has recently become one of the popular topics aiming to interpret and explain machine learning or deep learning models (Tjoa and Guan, 2021). Explainable AI is essential for users to understand and trust the model prediction results. It can help illustrate what the models perceive and explain how these perceptions can be mapped with the underlying knowledge of the human. Some of the favored approaches to obtain an explanation from a complex black-box model are LIME (Ribeiro et al., 2016) and SHAP (Lundberg and Lee, 2017). LIME builds a local surrogate model to explain individual prediction. SHAP (Shapley Additive exPlanations) introduced SHAP values representing the unified measure of feature importance together with SHAP value estimation methods. DeepSHAP (Chen et al., 2019) was built based on the connection between the original SHAP and DeepLIFT (Shrikumar et al., 2017) to explain the deep learning model and further refined and extended with relative background distributions and stacks of mixed model types.

With still some ambiguities in classifying lncRNA and mRNA sequences based on training features, together with the promising results of 1D-CNN in previous applications, in this paper, we propose Xlnc1DCNN, a 1D-CNN model for classifying lncRNA and mRNA with an explanation. The model solely uses nucleotide sequences as the training set. On the human test set, Xlnc1DCNN outperformed all other models in terms of accuracy and F1-score. For the cross-species dataset, Xlnc1DCNN also had the generalization across testing species. We explained how the Xlnc1DCNN distinguished the lncRNA from mRNA transcript sequences by applying DeepSHAP to generate SHAP values representing how the model captured and visualized the contribution of each nucleotide using an in-house python code. The explanation of true positives (i.e., lncRNA transcript sequences) showed that the model classified a sequence as lncRNA if the sequence did not contain any important regions or contained only an N-terminal signal peptide or transmembrane helices. The explanation of true negatives (i.e., mRNA transcript sequences) showed that the model learned protein domains/families from the input transcript sequences and used them to predict the sequences as mRNAs. The explanation of false positives (i.e., mRNA predicted as lncRNA transcript sequences) showed that the model could not capture any important regions representing protein domains/families or found important regions contributing to both lncRNA and mRNA prediction. A few false positive sequences were also found with inconsistent transcript types among the databases. Lastly, the explanation of false negatives (i.e., lncRNA predicted as mRNA transcript sequences) showed that the model captured protein domains or families within these lncRNA sequences and, hence, misclassified them as mRNAs.

## 2 Materials and Methods

### 2.1 Data Compilation and Pre-Processing

The human transcript datasets for training the model were obtained from GENCODE (Frankish et al., 2018) and LNCipedia (Volders et al., 2018). GENCODE (release 32) contains 48,351 sequences of lncRNA transcripts and 100,291 sequences of protein-coding transcripts (PCTs). For LNCipedia (version 5.2), only high confidence sequences were selected, which resulted in 107,039 lncRNA transcripts. To remove lncRNA transcript sequences from LNCipedia that are duplicates of GENCODE, we used CD-HIT-EST-2D (Li and Godzik, 2006) to compare lncRNA sequences between LNCipedia and GENCODE and filter out the sequences with more than 95% similarity from the LNCipedia dataset. A total of 72,803 lncRNA sequences from LNCipedia remained. We then pre-processed the sequences used for training the Xlnc1DCNN model by discarding the sequences shorter than 200 bases and longer than 3,000 bases. After filtering, one-hot encoding was used to encode the sequences. The total number of remaining sequences after cleansing was 185,030 with 108,578 lncRNAs and 76,453 PCTs (Table 1). The lncRNAs and PCTS were set as the positive and negative classes, respectively. The dataset was stratified split by 80% and 20% into the training and test sets.

**TABLE 1:** Summary of datasets from GENCODE and LNCipedia.

Sequence Type	Species	Data Source	Dataset Size	<200 bps	>3,000 bps	No.of Transcripts after Cleansing
mRNA	Human	GENCODE (release 32)	100,291	374	23,464	76,453
lncRNA	Human	GENCODE (release 32)	48,351	291	3,486	44,574
lncRNA	Human	LNCipedia (version 5.2)	72,803	0	8,799	64,004

Cross-species datasets included the mouse dataset obtained from GENCODE (Frankish et al., 2018) (release M23) and the gorilla, chicken, and cow datasets obtained from Ensembl (Cunningham et al., 2019) (release 102). We pre-processed the cross-species datasets by discarding the sequences shorter than 200 bases and longer than 3,000 bases. We then randomly selected the mRNA and lncRNA sequences for each species. The test transcripts of gorilla, chicken, cow, and mouse contained 8,000, 8,000, 11,000, and 32,000 sequences, respectively, each with an equal number of sequences from each class.

### 2.2 Model Architecture

In this study, we designed and implemented the Xlnc1DCNN model in Python3 using TensorFlow on NVIDIA GeForce GTX 1080 Ti and Intel Xeon Silver 4112 Processor. The built model could distinguish lncRNAs from the mRNAs (PCTs) and outperformed the existing tools for the human dataset. The model architecture consists of three convolutions with pooling layers, two fully connected layers, and a Softmax layer. We used ReLU as the activation function for convolution and fully connected layers. We also found that adding the dropout layer after the pooling layer made the model perform slightly better.

We used 10% of the data from the training set to perform hyperparameter optimizations over the kernel size, dropout rate, stride size, batch size, and learning rate by using the grid search algorithm. The best kernel size was 57, with the stride size equal to 1. The model performance started to decrease after increasing the stride size for almost every kernel size. For the learning details, the momentum, learning rate, number of epochs, and batch size were 0.9, 0.01, 120, and 128, respectively, with the stochastic gradient descent as an optimizer. The final hypermeters used in the model architecture are shown in Table 2.

**TABLE 2:** Hyperparameters of the proposed 1D-CNN architecture.

Layer	Hyperparameter
Conv 1D	kernel size = 57, stride = 1
Max-Pooling	pool size = 2
Dropout	*p* = 0.3
Conv 1D	kernel size = 57, stride = 1
Max-Pooling	pool size = 2
Dropout	*p* = 0.3
Conv 1D	kernel size = 57, stride = 1
Max-Pooling	pool size = 2
Dropout	*p* = 0.3
Flatten	-
Dense	256
Dropout	*p* = 0.5
Dense	256
Dropout	*p* = 0.5
Softmax	2

### 2.3 Model Interpretation

DeepSHAP was used to interpret how the proposed Xlnc1DCNN model could classify the lncRNAs and mRNAs from the input transcript sequences. As DeepSHAP needs background distributions as references to approximate the SHAPley values on conditional expectation, 175 sequences from each class were randomly selected as the representative background. A total of 350 sequences were used as the backgrounds as it was limited by the available GPU.

**FIGURE 1:**
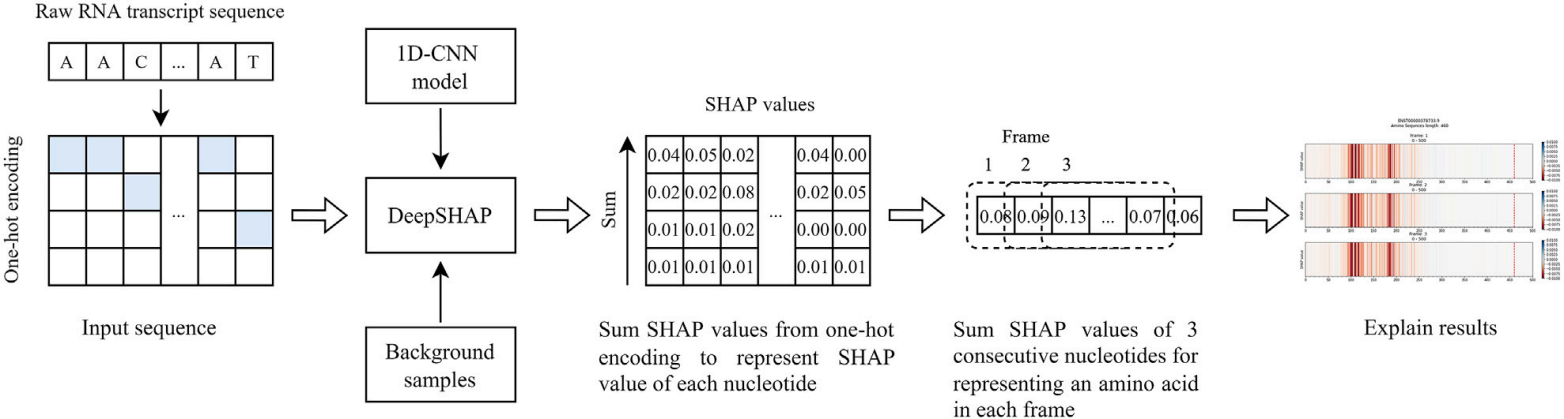
The process to obtain SHAP values for explaining the nucleotide contribution that was captured by the model to differentiate lncRNA from mRNA transcript sequences.

The output from DeepSHAP is SHAP values representing each nucleotide’s contribution to the model. To obtain SHAP values representing each nucleotide within a sequence, we summed up SHAP values inside the array of one-hot encoding and got a single SHAP value of each nucleotide. To visualize SHAP values from DeepSHAP of the input transcript sequence, we further summed up the SHAP values of three consecutive nucleotides, which probably represented an amino acid, and generated the results in three reading frames. We then plotted a color line for each representative amino acid. The blue and red colors, respectively, indicate the contribution of each amino acid for classifying the sequence as an lncRNA and an mRNA (Figure 1).

### 2.4 Evaluation

#### 2.4.1 Model Evaluation Metrics

To evaluate the performance of the proposed Xlnc1DCNN model with other existing tools, we used the following metrics. True positive (TP) represents the lncRNA transcript sequences that are predicted as lncRNAs. True negative (TN) represents PCTs that are predicted as PCTs. False positive (FP) represents the PCTs that are predicted as lncRNAs. False negative (FN) represents lncRNAs that are predicted as PCTs.

$$\mathrm{Accuracy}=\frac{\mathrm{TP}+\mathrm{TN}}{\mathrm{TP}+\mathrm{TN}+\mathrm{FP}+\mathrm{FN}}$$

$$\mathrm{Sensitivity}=\frac{\mathrm{TP}}{\mathrm{TP}+\mathrm{FN}}$$

$$\mathrm{Specificity}=\frac{\mathrm{TN}}{\mathrm{TN}+\mathrm{FP}}$$

$$\mathrm{Precision}=\frac{\mathrm{TP}}{\mathrm{TP}+\mathrm{FP}}$$

$$F1-\mathrm{Score}=\frac{2\times \mathrm{precision}\times \mathrm{sensitivity}}{\mathrm{precision}+\mathrm{sensitivity}}$$

#### 2.4.2 Interpretation Evaluation Method

To compare the explanation results of Xlnc1DCNN on the human test set with known biological knowledge, we utilized the available bioinformatics tools/databases such as TMHMM (Krogh et al., 2001) to identify transmembrane helices, Pfam (Mistry et al., 2020), and InterPro (Blum et al., 2020) to identify protein domains or families for all sequences in the test set. From InterPro, we considered InterPro entries, which include InterPro domain, family, homologous superfamily, repeat, and sites (i.e., active site, binding site, conserved site, PTM site). MobiDB (integrated within InterPro) (Piovesan et al., 2021) was also used to identify intrinsically disordered regions within sequences.

## 3 Results

### 3.1 Model Evaluation Results

We compared the performance of Xlnc1DCNN with eight existing tools: CPC2, CPAT, CNIT, PLEK, FEELnc, RNAsamba, LncADeep, and lncRNA_Mdeep (Wang et al., 2013; Li et al., 2014; Kang et al., 2017; Wucher et al., 2017; Yang et al., 2018; Guo et al., 2019; Camargo et al., 2020; Fan et al., 2020) with the version listed in Supplementary Table S1. To have a fair and unbiased evaluation, we retrained CPAT, FEELnc, and RNAsamba that provided a training option using our human training dataset and used the pre-trained models of CPC2, CNIT, and LncADeep that did not provide a training option. Although PLEK and lncRNA_Mdeep came with a training option, retraining PLEK and lncRNA_Mdeep was very time-consuming, so we skipped retraining both and used their default pre-trained models.

#### 3.1.1 Performance Evaluation on the Human Test Set

**FIGURE 2:**
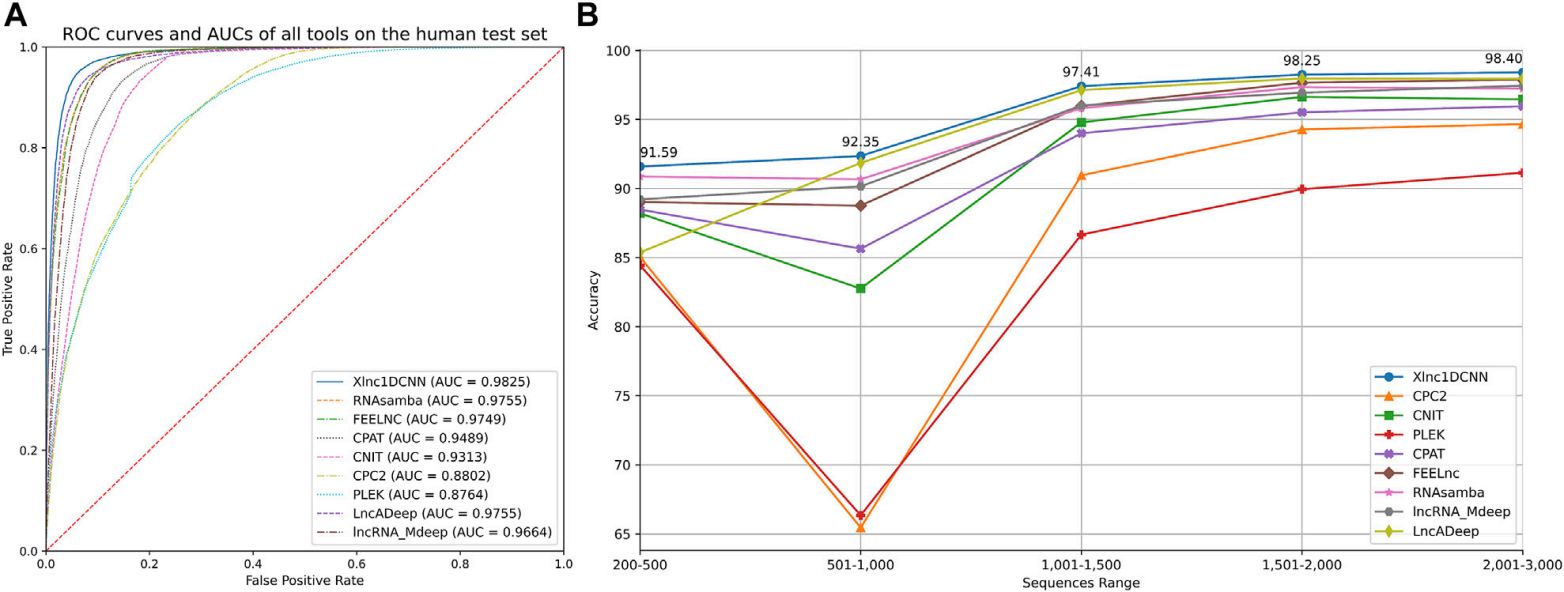
**(A)** ROC curves of all tools and their AUCs on the human test set. **(B)** Accuracy of all tools for any range of sequence lengths of the human test set.

The results on the human test set (Table 3) show that Xlnc1DCNN achieved the highest accuracy (94.53) and F1-Score (95.38), the second-highest precision (94.55) slightly lower than LncADeep, and the third-highest specificity (92.13) slightly lower than LncADeep and FEELnc. CPC2, CNIT, and CPAT achieved high sensitivity but much lower specificity. While FEELnc, RNAsamba, LncADeep, and lncRNA_Mdeep performed well on the average of every metric but overall, still lower than Xlnc1DCNN. We then analyzed the classification power of each tool by plotting a receiver operating characteristic curve (ROC) and measuring the area under the curve (AUC) as shown in Figure 2A, where Xlnc1DCNN achieved the highest AUC (0.9825) on the human test set. Figure 2B shows that Xlnc1DCNN also outperformed all tools on any range of sequence lengths of the human test set (Supplementary Table S2).

**TABLE 3:** Evaluation results of all tools on the human test set.

Model	TP	FP	TN	FN	Accuracy	Sensitivity	Specificity	Precision	F1
Xlnc1DCNN	20,895	1,204	14,087	821	**94.53**	96.22	92.13	94.55	**95.38**
CPC2	21,023	6,457	8,834	693	80.68	96.81	57.77	76.50	85.47
CNIT	21,307	3,580	11,711	409	89.22	**98.12**	76.59	85.61	91.44
PLEK	20,704	6,665	8,626	1,012	79.26	95.34	56.41	75.65	84.36
CPAT	20,646	2,597	12,694	1,070	90.09	95.07	83.02	88.83	91.84
FEELNC	20,023	1,182	14,109	1,693	92.23	92.20	92.27	94.43	93.30
RNASAMBA	20,998	1,795	13,496	718	93.21	96.69	88.26	92.12	94.35
lncRNA_Mdeep	20,813	1,799	13,492	903	92.70	95.84	88.23	92.04	93.90
LncADeep	20,232	1,113	14,178	1,484	92.98	93.17	**92.72**	**94.79**	93.97

The bold values indicate the highest value within each column.

#### 3.1.2 Performance Evaluation on Cross-Species Datasets

**FIGURE 3:**
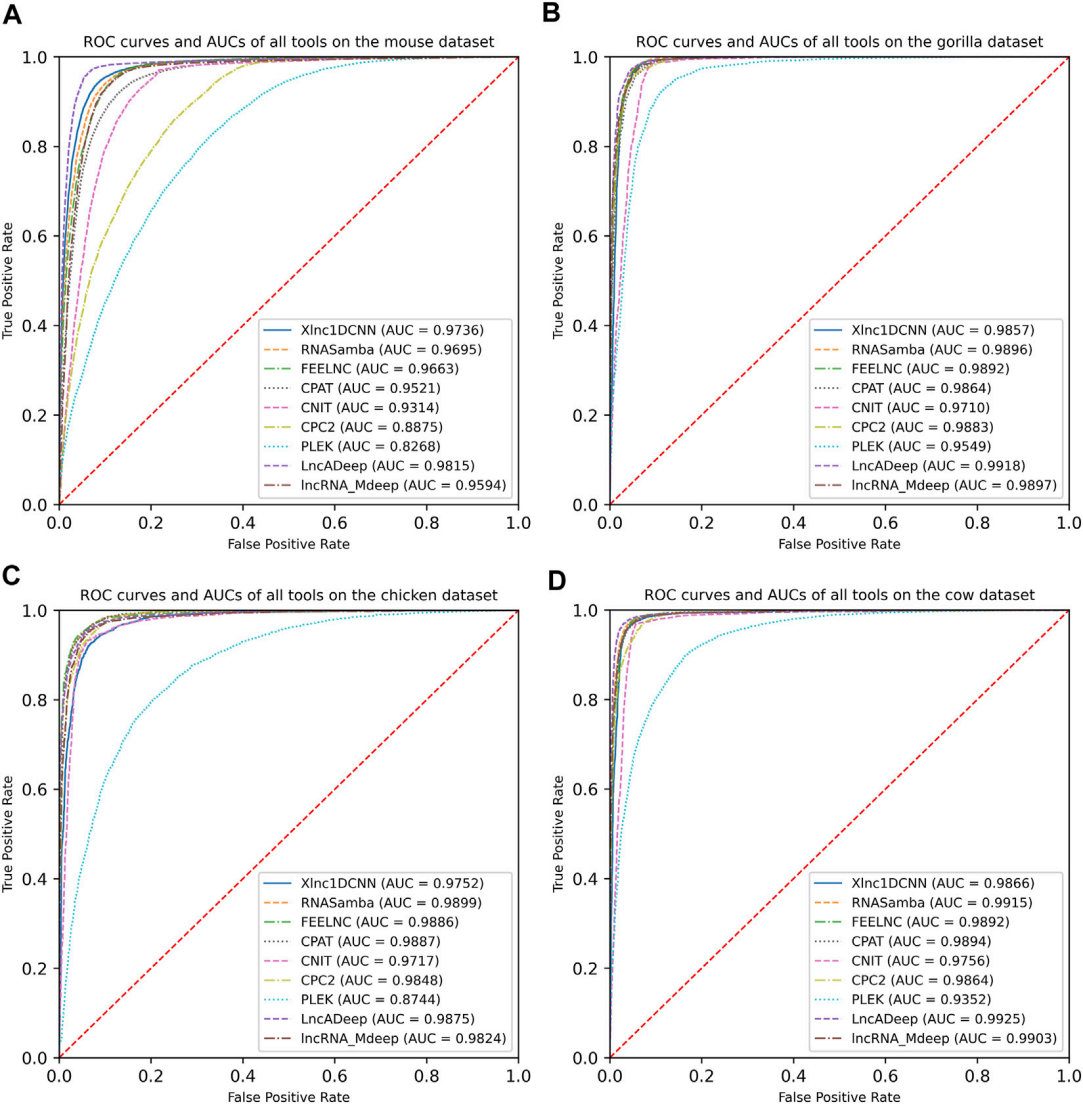
Receiver operating characteristic curves and AUCs of nine models on the datasets of **(A)** mouse, **(B)** gorilla, **(C)** cow, and **(D)** chicken.

To evaluate the generalization of Xlnc1DCNN with cross-species datasets, we compared the model with other tools using the mouse, gorilla, chicken, and cow datasets. The evaluation results show that Xlnc1DCNN, which was trained on the human dataset, has a generalization for classifying lncRNAs and mRNAs on other species (Table 4 and Supplementary Tables S3–S6). Xlnc1DCNN achieved the highest accuracy on the gorilla dataset together with RNAsamba and the second highest accuracy on the mouse dataset while LncADeep achieved the highest accuracy on mouse and cow datasets. Figure 3 shows that Xlnc1DCNN has the ROC curves and AUCs close to other tools on cross-species datasets. Overall, based on AUCs, LncADeep got the best generalization performance on cross-species datasets.

**TABLE 4:** Accuracy of the nine models on cross-species datasets.

Model	Mouse	Gorilla	Chicken	Cow
Xlnc1DCNN	92.58	**96.06**	92.35	95.92
CPC2	80.06	94.96	93.51	94.48
CNIT	87.68	94.00	92.94	95.18
PLEK	73.62	89.53	79.54	86.22
CPAT	89.46	95.1	93.70	95.52
FEELnc	90.51	94.8	92.75	93.97
RNAsamba	91.91	**96.06**	**93.98**	96.39
LncADeep	**94.95**	96.05	93.46	**96.70**
lncRNA_Mdeep	91.38	95.58	92.59	95.63

The bold values indicate the highest value within each column.

### 3.2 Model Interpretation Results

As Xlnc1DCNN outperformed other tools on the human test set, we assumed that 1D-CNN captured patterns within sequences that could be used to distinguish lncRNAs from mRNAs. To explain the model, we used DeepSHAP to describe the contribution of each nucleotide to the prediction results. The explanation output from DeepSHAP was SHAP values for all nucleotides of the entire sequence. This explanation result was then visualized based on the summed SHAP values of each three consecutive nucleotides, with important representative amino acids highlighted in the sequence.

In the following subsections, we present the explanation results of Xlnc1DCNN focusing on the true positive, true negative, false positive, and false negative sequences predicted by Xlnc1DCNN on the human test set.

#### 3.2.1 True Positive Sequences

**FIGURE 4:**
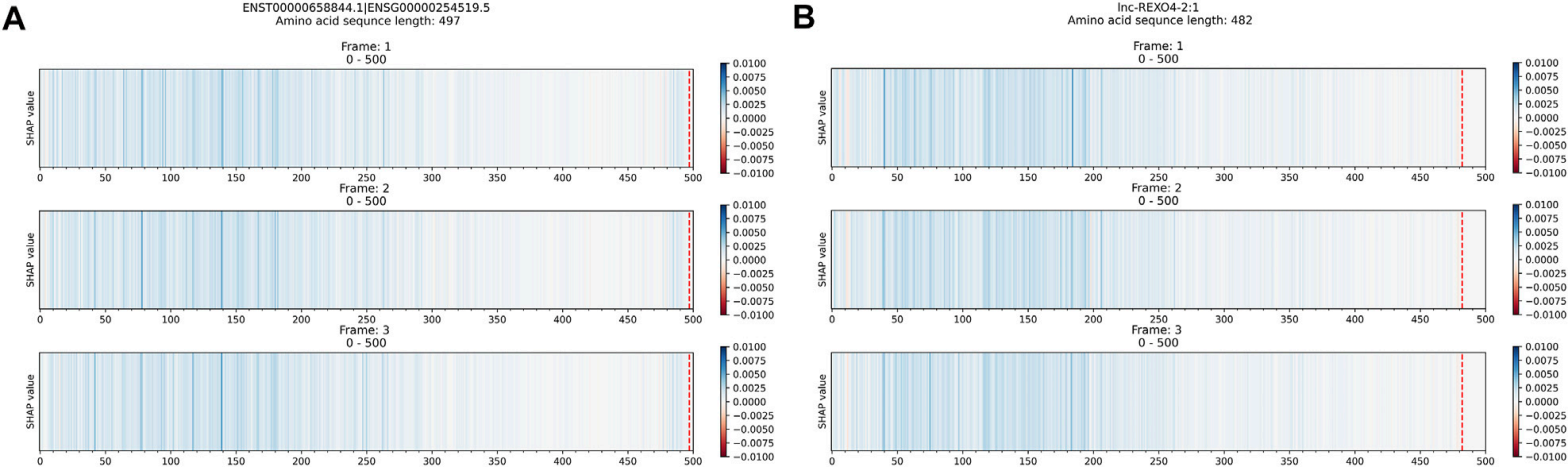
Explanation results of Xlnc1DCNN on TP sequences **(A)** ENST00000658844.1, a lncRNA sequence obtained from GENCODE and **(B)** lnc-REXO4-2:1, a lncRNA sequence obtained from LNCipedia.

The explanation results of Xlnc1DCNN highlighted the important regions that contributed to the correct classification of an input lncRNA transcript sequence as a lncRNA with blue color. From Figures 4A,B, the explanation results of the ENST00000658844.1 and lnc-REXO4-2:1 suggested that Xlnc1DCNN classified a transcript sequence as a lncRNA if it did not capture any important regions or specific patterns within the sequence. Additional explanation results of the TP sequences are shown in Supplementary Figures S1–S8.

#### 3.2.2 True Negative Sequences

**FIGURE 5:**
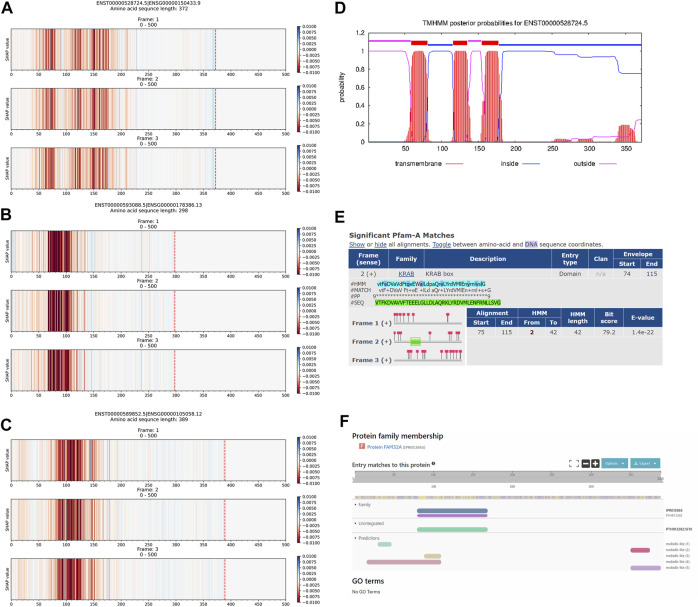
Comparison between the explanation results of Xlnc1DCNN on TN sequences **(A)** ENST00000528724.5 **(B)** ENST00000593088.5, and **(C)** ENST00000589852.5 protein-coding transcripts; and **(D)** prediction result of the TMHMM program on the ENST00000528724.5, **(E)** KRAB (Krüppel associated box) domain identified by Pfam within the ENST00000593088.5, and **(F)** FAM32A family identified by InterPro within the ENST00000589852.5 transcripts.

The explanation results of Xlnc1DCNN highlighted the important regions of a protein-coding transcript (i.e., mRNA) as red, as shown in Figures 5A–C. Figure 5D shows the transmembrane helix regions of the ENST00000528724.5 transcript predicted by TMHMM, corresponding to the important regions captured by Xlnc1DCNN. The prediction results of TMHMM and the explanation results of Xlnc1DCNN have similar patterns in several other mRNA transcripts within the test set (Supplementary Figures S9 and S10). Figure 5E shows the KRAB box (Krüppel associated box) identified by Pfam within the transcript ENST00000593088.5, which mostly overlapped with the important region captured by Xlnc1DCNN as shown in Figure 5B. Figure 5F shows the FAM32A family (family with sequence similarity 32 member A) identified by InterPro within the ENST00000589852.5 transcript, which corresponds to the important region of the ENST00000589852.5 identified by Xlnc1DCNN as shown in Figure 5C. This transcript has been linked to an ovarian tumor-associated gene (Chen et al., 2011). Additional explanation results of the TN sequences are shown in Supplementary Figures S11–S14.

#### 3.2.3 False Positive Sequences

**FIGURE 6:**
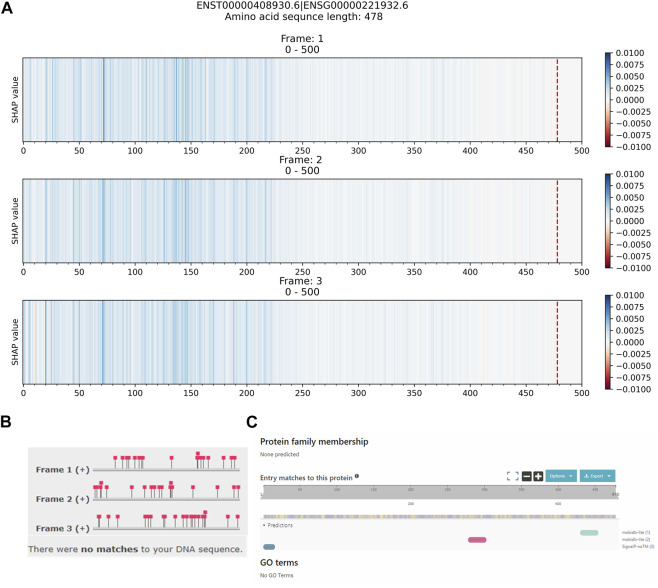
Comparison between **(A)** the explanation result of Xlnc1DCNN on the ENST00000408930.6 protein-coding transcript, predicted as a lncRNA, **(B)** identification result from Pfam, and **(C)** identification result from InterPro.

False positive sequences are mRNA transcript sequences that are predicted as lncRNAs. Figure 6A shows the explanation result of ENST00000408930.6, which did not contain any important regions with red color contributing to the prediction as an mRNA. Figures 6B,C show Pfam and InterPro’s results that both could not identify any protein domains or families within the ENST00000408930.6 protein-coding transcript. While the Ensembl database reports the ENST00000408930.6 as a protein-coding transcript of the HEPN1 (ENSG00000221932) gene, the Gene database at NCBI reports HEPN1 as the ncRNA gene (https://www.ncbi.nlm.nih.gov/gene/641654) and the RefSeq database reports the NR_170,124.1 (ENST00000408930.6) as a long non-coding RNA (https://www.ncbi.nlm.nih.gov/nuccore/NR_170124.1). Based on our evaluation, the top five long non-coding RNA identification (our Xlnc1DCNN, RNAsamba, LncADeep, lncRNA_Mdeep, FEELnc) predicted this sequence as lncRNA. This sequence highlights an example of inconsistent annotations among public databases that affect the model performance and evaluation. Additional explanation results of the FP sequences are shown in Supplementary Figures S15–S19.

#### 3.2.4 False Negative Sequences

**FIGURE 7:**
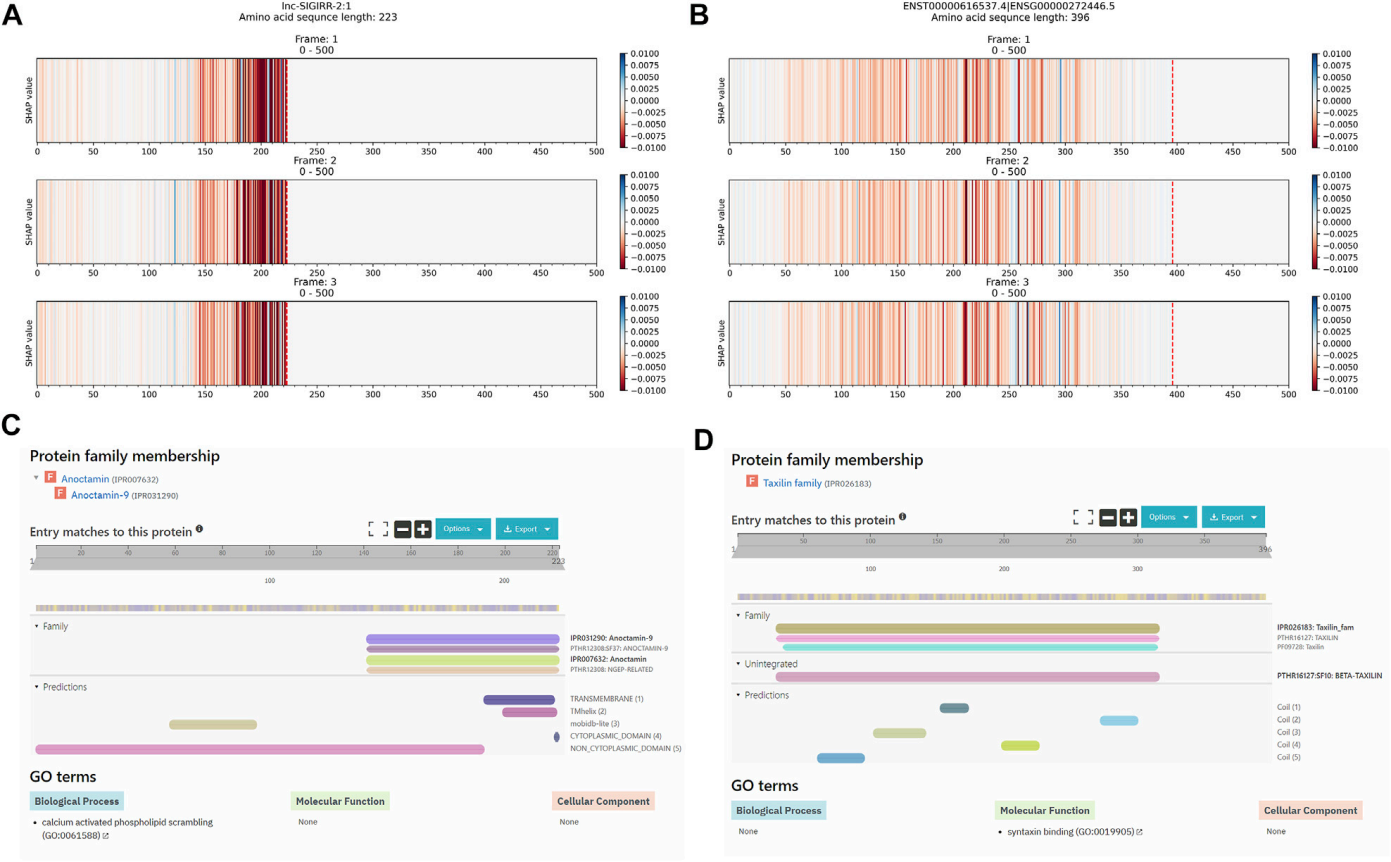
Comparison between the explanation result of Xlnc1DCNN on the long non-coding RNA transcripts **(A)** lnc-SIGIRR-2:1 and **(B)** ENST00000616537.4, predicted as mRNAs; **(C)** Anoctamin family within the lnc-SIGIRR-2:1 transcript and **(D)** Taxilin family within the ENST00000616537.4 transcript identified by InterPro.

False negative sequences are lncRNA transcript sequences that are predicted as mRNAs. Figures 7A,B show the explanation results of lncRNAs: LNC-SIGIRR-2:1 and ENST00000616537.4 with important regions that contributed to the wrong prediction as mRNA transcripts. These regions correspond to the identified Anoctamin and the Taxilin InterPro families identified by InterPro, as shown in Figures 7C,D. Additional explanation results of the FN sequences are shown in Supplementary Figures S20–S25.

## 4 Discussion

The explanation results of Xlnc1DCNN on the true positive sequences (TPs) show that most of the lncRNAs were found with no conserved regions or patterns in short regions with unknown functions, i.e., the highlighted regions do not correspond to any InterPro entries (Supplementary Figures S1, S2). The important regions of some other lncRNA sequences highlighted transmembrane helices (Supplementary Figures S3–S5) or signal peptides (Supplementary Figures S6–S8). Over recent years, some studies also found a transmembrane helix inside lncRNAs (Anderson et al., 2015; Makarewich, 2020) and hidden peptides encoded within non-coding RNAs (Matsumoto and Nakayama, 2018). These findings correspond to what Xlnc1DCNN has learned and highlighted via the explanation result as important regions for classifying a sequence as lncRNA. Out of 20,895 TPs, only 1,692 (8.10%) TPs were found with InterPro entries, 9,833 (47.06%) TPs were found with only intrinsically disordered regions (IDRs), and 11,490 (36.91%) TPs were found with transmembrane helices identified by TMHMM without any InterPro entries. Although 8.10% of TPs were found with InterPro entries, top protein domains and families of the TPs were found in only a few TNs (≤5) on the test set (Supplementary Tables S7, S8).

On the true negative sequences (TNs), the explanation results of Xlnc1DCNN show that the model could capture the regions representing the protein domains or families in the transcript sequences. Out of 14,087 TNs, 13,079 (92.86%), 882 (5.84%), and 289 (2.05%) TNs were found with InterPro entries, only IDRs, and transmembrane helices were identified by TMHMM without any InterPro entries. Hence, it could classify most of the input mRNA sequences correctly as the protein-coding transcripts.

The explanation results of false positive sequences (FPs) typically do not contain the important regions (red color) that contributed to the model prediction as mRNAs. Out of 1,204 FPs, 500 (42.53%) FPs were found without any InterPro entries, 359 (29.81%) FPs were found with only IDRs, and 161 (13.37%) FPs were found with transmembrane helices without any InterPro entries.

For false negative sequences (FNs), from a total of 821 FNs, there were 463 (56.39%) FNs found with InterPro entries, and the explanation results of FNs also correspond to these entries as shown in Figure 7, and Supplementary Figures S20–S25, 264 (32.16%) FNs were found with only IDRs and 104 (12.67%) FNs were found with transmembrane helices.

We summarized the TP, TN, FP, and FN sequences of the test set annotated with InterPro entries in Table 5. For TPs, most of the sequences were found without InterPro entries, in contrast with TNs. The number of TPs annotated with only IDRs, or transmembrane helices also highlighted the contributions of these regions to the predicted sequences as lncRNAs. The 704 out of 1,204 (58.47%) and 358 out of 821 (43.61%) annotated FPs and FNs with and without InterPro entries indicated the limitations of Xlnc1DCNN. We then further analyzed the misclassified FPs and FNs by top tools (Xlnc1DCNN, RNAsamba, LncADeep lncRNA_Mdeep, FEELnc). The 93 out of 344 (27.03%) and 15 out of 105 (14.92%) annotated FPs and FNs with and without InterPro entries misclassified by all top tools suggested sequences that were difficult to identify. Finally, the 251 out of 344 (72.97%) and 90 out of 105 (85.71%) annotated FPs and FNs without and with InterPro entries misclassified by all top tools suggested the possible limitations of all top tools or inconsistent annotations across the public databases.

**TABLE 5:** Summary of test set sequences annotated with InterPro entries.

Metrics	Amount	Found with InterPro Entries	Found without InterPro Entries	Contain IDRs without InterPro Entries	Contain Transmembrane Helices without InterPro Entries
TP	20,895	1,692 (8.10%)	19,203 (91.9%)	9,833 (47.06%)	7,713 (36.91%)
TN	14,087	13,085 (92.89%)	1,002 (7.11%)	822 (5.84%)	289 (2.05%)
FP	1,204	704 (58.47%)	500 (41.53%)	359 (29.82%)	161 (13.37%)
FN	821	463 (56.39%)	358 (43.61%)	264 (32.16%)	104 (12.67%)
All missed FP	344	93 (27.03%)	251 (72.97%)	164 (47.67%)	94 (27.33%)
All missed FN	105	90 (85.71%)	15 (14.92%)	5 (4.76%)	4 (3.81%)

**FIGURE 8:**
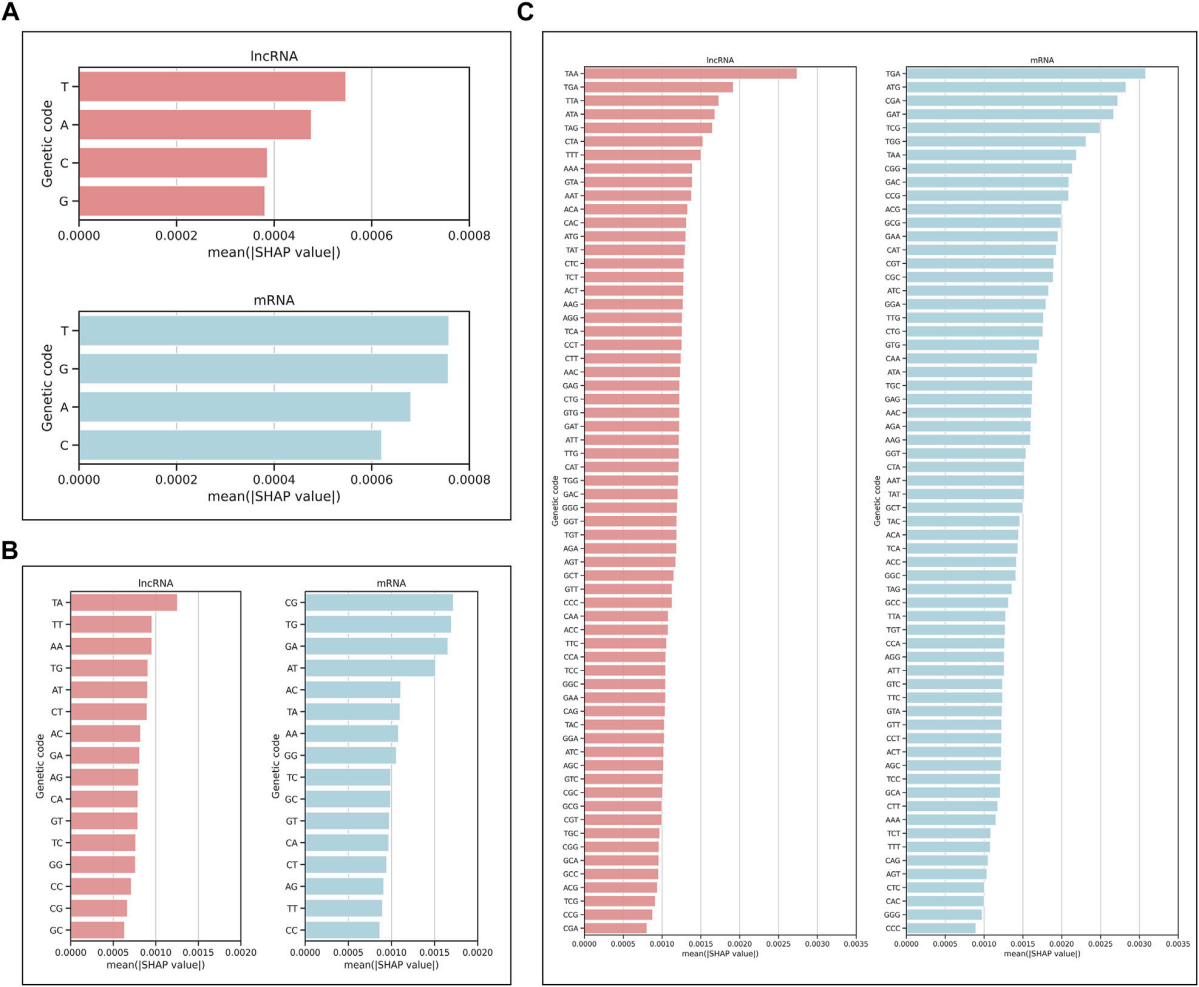
Mean of absolute SHAP values for **(A)** single nucleotide, **(B)** dinucleotide, and **(C)** trinucleotide, indicating the impact of each genetic code on the model prediction as lncRNA or mRNA.

We also analyzed the contribution of each nucleotide by plotting the mean of absolute SHAP values on the test set for a single nucleotide, dinucleotide, and trinucleotide (codon). The higher mean of absolute SHAP values indicates the higher impact of that genetic code (Figure 8). For lncRNA, we found that the top three codons with the highest contribution were all stop codons (TAA, TGA, TTA), and for mRNA, the top three were the stop codon, start codon, and arginine (TGA, ATG, CGA). For dinucleotide, CG has the highest mean of absolute SHAP values for classifying as mRNA, which is consistent with those of (Ulveling et al., 2014).

As recent studies found that some putative lncRNAs contain a short open reading frame (sORF) (Hartford and Lal, 2020), we further analyzed the association of lncRNAs and sORF using the explanation results of Xlnc1DCNN. Some false negative sequences were randomly selected and checked if they contained sORF using MetamORF (Choteau et al., 2021). While MetamORF found sORFs in some of these sequences, the reported regions of these sORFs did not correspond to the important regions highlighted by the explanation results.

## 5 Conclusion

In this study, we proposed Xlnc1DCNN, a simple but effective 1D-CNN model for classifying and explaining lncRNA and protein-coding transcripts. We have shown that using 1D-CNN as a feature extractor can lead to a better prediction performance than other existing tools using traditional feature extraction methods. The explanation results provided insights into what the model learned to distinguish the lncRNA from protein-coding transcripts. The transmembrane helix region highlighted by the explanation results of several true positive lncRNA transcripts agreed with the recent findings of transmembrane microproteins within lncRNAs. Disordered proteins without any important regions highlighted in the explanation results were misclassified as lncRNAs. Several explanation results of lncRNA misclassified as protein-coding transcripts contained important regions that correspond to protein domains or families in Pfam and/or InterPro. These insights revealed the complexity of long non-coding RNAs and the need to evaluate cross-referenced gene annotation among public databases periodically.

## Data Availability

The original contributions presented in the study are included in the article/Supplementary Materials, further inquiries can be directed to the corresponding author.
