# Supplementary material for: Interpretable Deep Learning Model Reveals Subsequences of Various Functions for Long Non-Coding RNA Identification
Source: Front Genet. 2022 May 24;13:876721. doi: 10.3389/fgene.2022.876721 (PMC9173695; doi:10.3389/fgene.2022.876721)
Supplement: Supplementary file 1 [file DataSheet1.PDF]

Supplementary Material

Table S1. Software version.

Software	Version
CPC2	Web based
CNIT	Web based
PLEK	1.2
CPAT	2.0.0
FEELnc	0.1.1
RNAsamba	0.2.4
LncADeep	1.0
lncRNA_Mdeep	-

Table S2. The number of mRNA and lncRNA transcripts of the human test set varied by sequence lengths.

Sequence lengths (bps)	Number of mRNA sequences	Number of lncRNA sequences
200–500	1,149	6,049
501–1,000	6,666	7,914
1,001–1,500	2,124	3,438
1,501–2,000	2,189	2,090
2,000–3,000	3,163	2,225

Table S3. Evaluation results of all tools on gorilla transcripts.

Model	TP	FP	TN	FN	Accuracy	Sensitivity	Specificity	Precision	F1-Score
Xlnc1DCNN	3,902	217	3,783	98	96.06	97.55	94.58	94.73	96.12
CPC2	3,878	281	3,719	122	94.96	96.95	92.98	93.24	95.06
CNIT	3,944	424	3,576	56	94.00	98.60	89.40	90.29	94.26
PLEK	3,847	685	3,315	153	89.53	96.18	82.88	84.89	90.18
CPAT	3,824	216	3,784	176	95.10	95.60	94.60	94.65	95.12
FEELnc	3,723	139	3,861	277	94.80	93.08	96.53	96.40	94.71
RNAsamba	3,899	214	3,786	101	96.06	97.48	94.65	94.80	96.12
lncRNA_Mdeep	3,863	217	3,783	137	95.58	96.58	94.58	94.68	95.62
LncADeep	3,842	158	3,842	158	96.05	96.05	96.05	96.05	96.05

Table S4. Evaluation results of all tools on chicken transcripts.

Model	TP	FP	TN	FN	Accuracy	Sensitivity	Specificity	Precision	F1-Score
Xlnc1DCNN	3,606	218	3,782	394	92.35	90.15	94.55	94.30	92.18
CPC2	3,679	198	3,802	321	93.51	91.98	95.05	94.89	93.41
CNIT	3,737	302	3,698	263	92.94	93.43	92.45	92.52	92.97
PLEK	3,119	756	3,244	881	79.54	77.98	81.10	80.49	79.21
CPAT	3,593	97	3,903	407	93.70	89.83	97.58	97.37	93.45
FEELnc	3,485	65	3,935	515	92.75	87.13	98.38	98.17	92.32
RNAsamba	3,618	100	3,900	382	93.98	90.45	97.50	97.31	93.75
lncRNA_Mdeep	3,538	131	3,869	462	92.59	88.45	96.73	96.43	92.27
LncADeep	3,586	109	3,891	414	93.46	89.65	97.28	97.05	93.20

Table S5. Evaluation results of all tools on mouse transcripts.

Model	TP	FP	TN	FN	Accuracy	Sensitivity	Specificity	Precision	F1
Xlnc1DCNN	15,307	1,680	14,320	693	92.58	95.67	89.50	90.11	92.81
CPC2	15,186	5,568	10,432	814	80.06	94.91	65.20	73.17	82.64
CNIT	15,530	3,473	12,527	470	87.68	97.06	78.29	81.72	88.74
PLEK	14,731	7,172	8,828	1,269	73.62	92.07	55.18	67.26	77.73
CPAT	14,812	2,186	13,814	1,188	89.46	92.58	86.34	87.14	89.78
FEELnc	14,244	1,281	14,719	1,756	90.51	89.03	91.99	91.75	90.37
RNAsamba	15,161	1,749	14,251	839	91.91	94.76	89.07	89.66	92.14
lncRNA_Mdeep	14,858	1,616	14,384	1,142	91.38	92.86	89.90	90.19	91.51
LncADeep	15,388	1,003	14,997	612	94.95	96.18	93.73	93.88	95.01

Table S6. Evaluation results of all tools on cow transcripts.

Model	TP	FP	TN	FN	Accuracy	Sensitivity	Specificity	Precision	F1
Xlnc1DCNN	5,259	208	5,292	241	95.92	95.62	96.22	96.20	95.91
CPC2	5,201	308	5,192	299	94.48	94.56	94.40	94.41	94.49
CNIT	5,324	354	5,146	176	95.18	96.80	93.56	93.77	95.26
PLEK	4,751	767	4,733	749	86.22	86.38	86.05	86.10	86.24
CPAT	5,194	187	5,313	306	95.52	94.44	96.60	96.52	95.47
FEELnc	4,491	124	5,376	509	93.97	89.82	97.75	97.31	93.42
RNAsamba	5,294	191	5,309	206	96.39	96.25	96.53	96.52	96.39
lncRNA_Mdeep	5,188	169	5,331	312	95.63	94.33	96.93	96.85	95.57
LncADeep	5,262	121	5,379	238	96.74	95.67	97.80	97.75	96.70

Table S7. Top protein domains found within TPs compared with TNs.

Protein Domain	Found in TPs	Found in TNs
Murine leukemia virus integrase, C-terminal	30	1
Domain of unknown function DUF1725	29	3
Reverse transcriptase domain	17	2
L1 transposable element, dsRBD-like domain	13	1
L1 transposable element, RRM domain	13	0
NADH:quinone oxidoreductase/Mrp antiporter, membrane subunit	13	0
Ribosomal protein S10 domain	9	5
Mos1 transposase, HTH domain	9	1
Retro-transcribing virus envelope glycoprotein	9	1
Integrase, catalytic core	8	1
DDE superfamily endonuclease domain	7	3
Ribosomal protein L23/L25, N-terminal	6	1
Cytochrome c-like domain	6	1
Ribosomal protein L30, ferredoxin-like fold domain	5	3
Domain of unknown function DUF4764	5	1
Reverse transcriptase/retrotransposon-derived protein, RNase H-like domain	5	0
Mitochondrial cytochrome c oxidase subunit VIc/VIIs	5	1
Cytochrome c oxidase subunit II-like C-terminal	5	0
Integrase, C-terminal, retroviral	5	3
Cytochrome b/b6, N-terminal	5	0

Table S8. Top protein families found within TPs compared with TNs.

Protein Family	Found in TPs	Found in TNs
TLV/ENV coat polyprotein	23	2
Ribosomal protein L21e	18	1
High mobility group protein HMGN	16	5
BNIP3	11	3
Transposase, L1	11	0
Ribosomal protein L44e	10	2
Ribosomal protein S26e	10	0
Ribosomal protein S10	10	2
Ribosomal protein L34Ae	9	0
Ribosomal protein S27	7	2
Ribosomal protein S12e	7	0
Ribosomal protein S8	7	2
Vomeronasal receptor, type 1	7	0
Transposase, type 1	7	0
High mobility group protein HMGB1	7	0
FAM27D/FAM27E	6	0
Protein FAM27	6	0
NADH-ubiquinone reductase complex 1 MLRQ subunit	6	1
Elongin-C	6	4
Ribosomal protein S3Ae	6	2

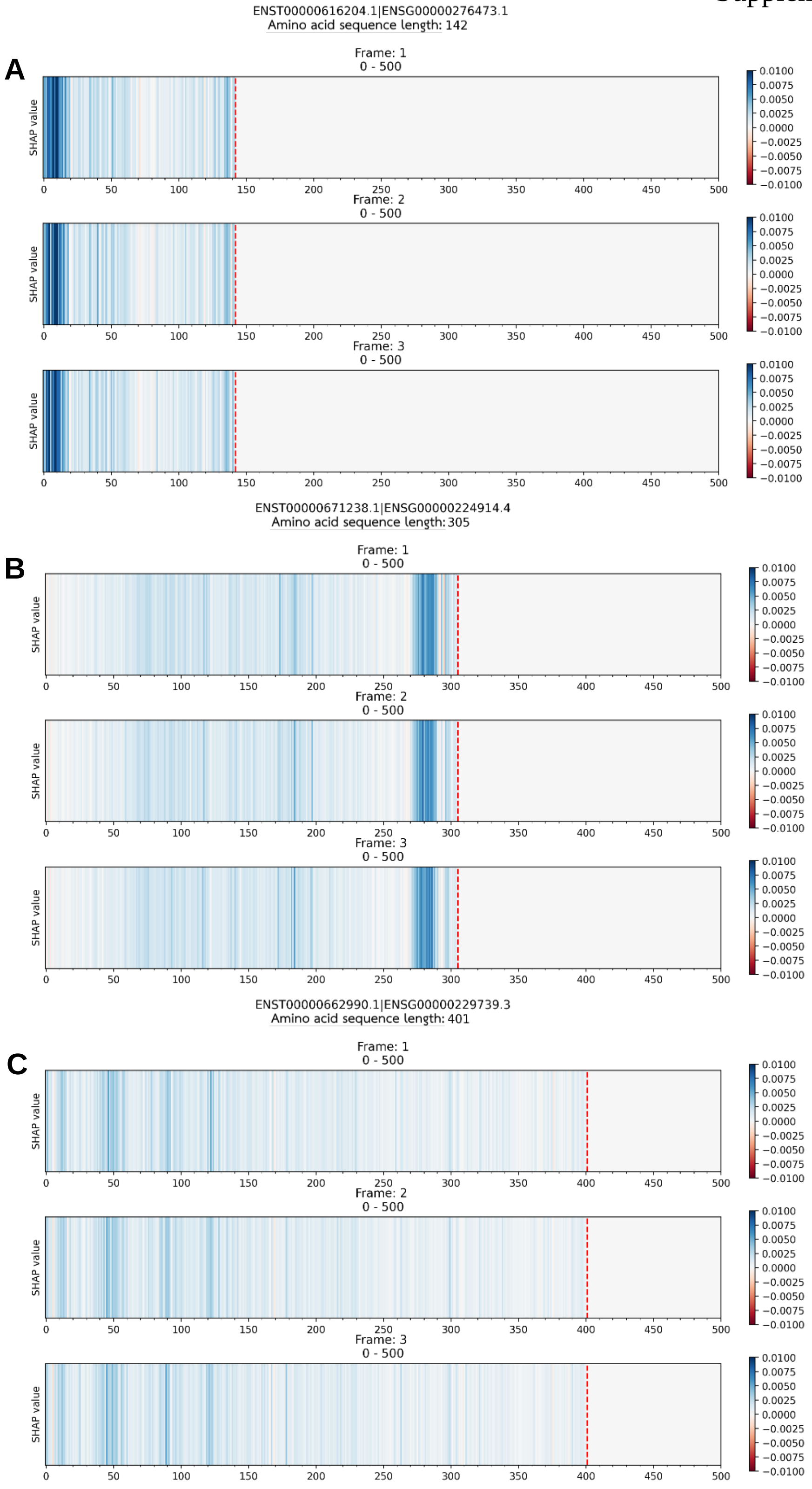

Figure S1. The explanation results of Xlnc1DCNN on true positive sequences of the (**A**) ENST00000616204.1, (**B**) ENST00000671238.1, and (**C**) ENST00000662990.1 obtained from GENCODE.

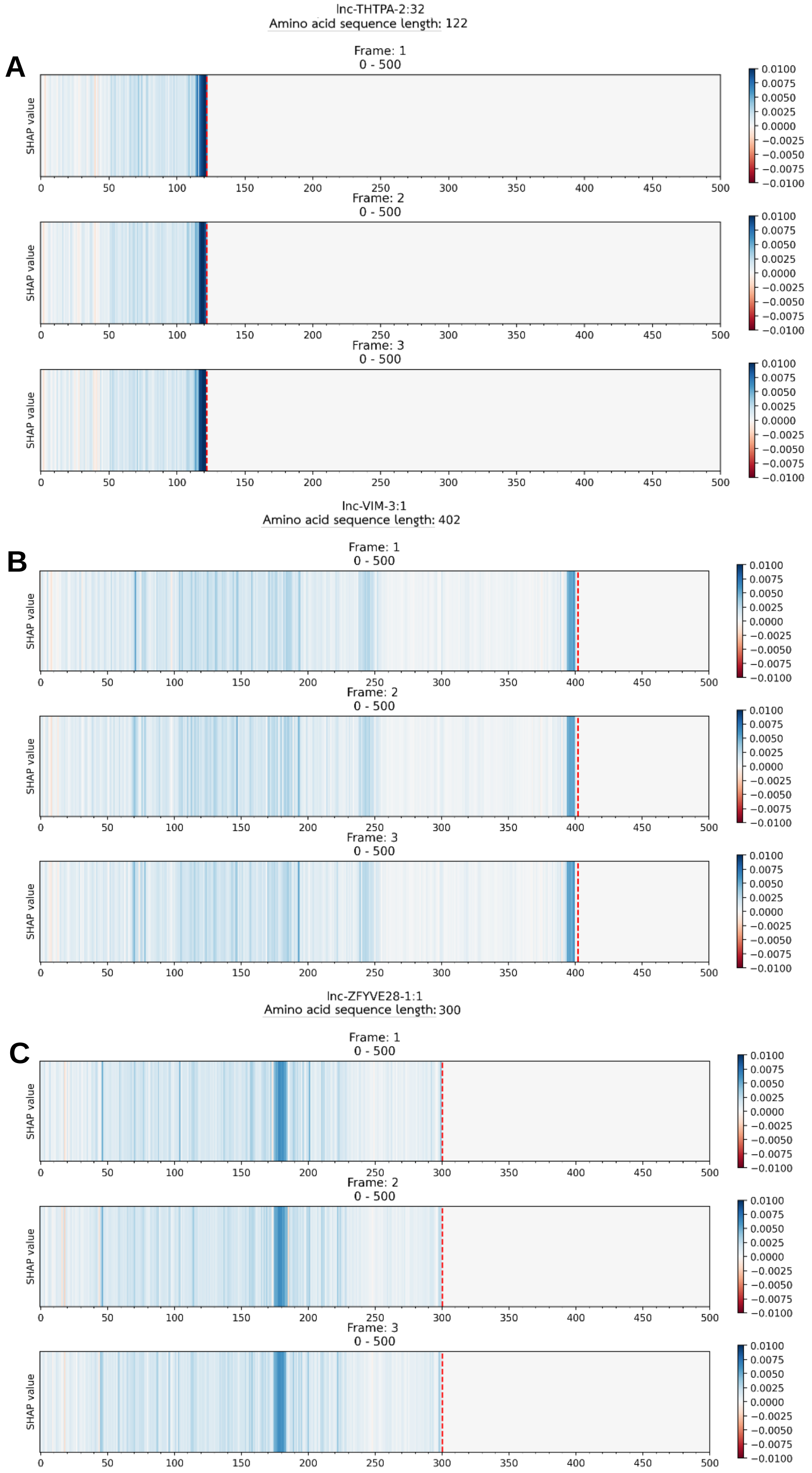

Figure S2. The explanation results of Xlnc1DCNN on true positive sequences of the (**A**) lnc-THTPA-2:32, (**B**) lnc-VIM-3:1, and (**C**) lnc-ZFYVE28-1:1 obtained from LNCipedia.

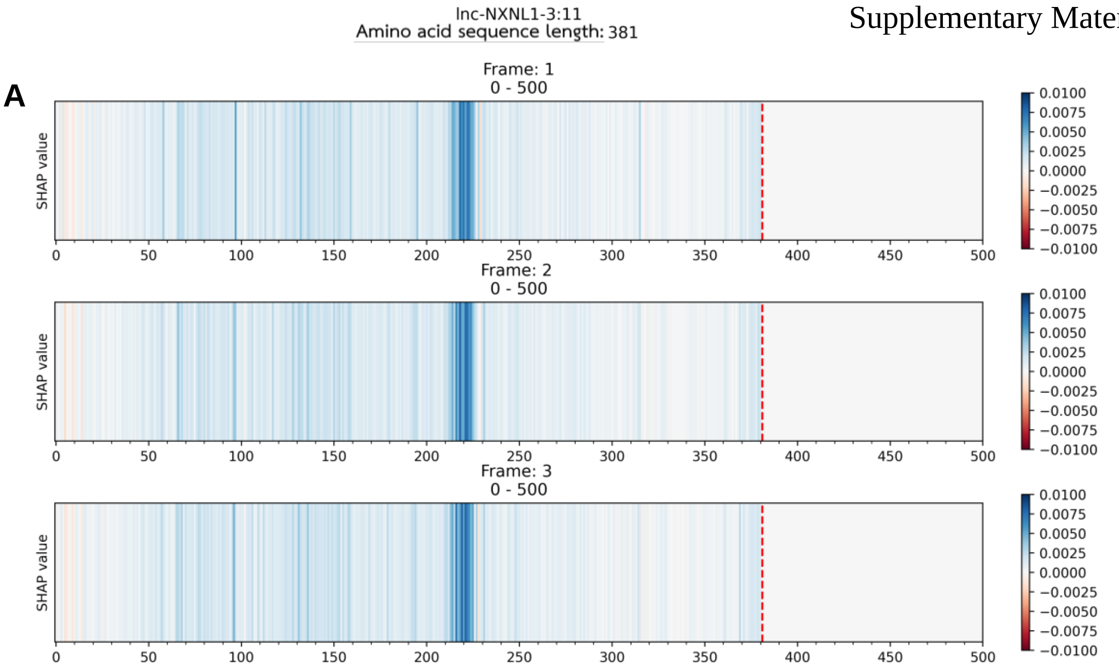

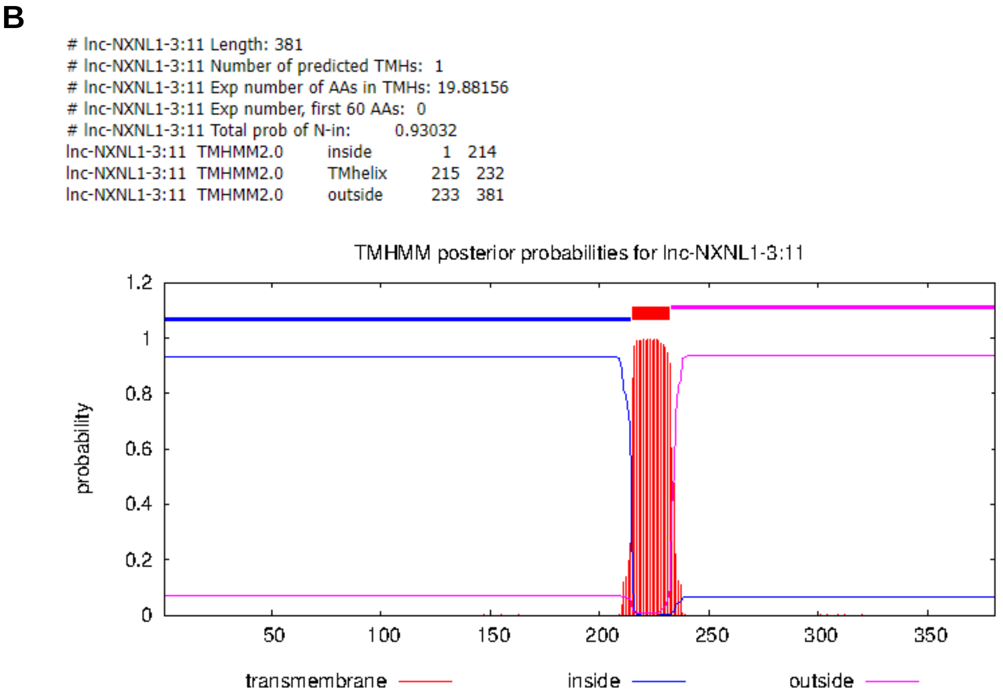

Figure S3. (**A**) The explanation result of Xlnc1DCNN and (**B**) the prediction result of the transmembrane helices by TMHMM program on the true positive sequence, lnc-NXNL1-3:11.

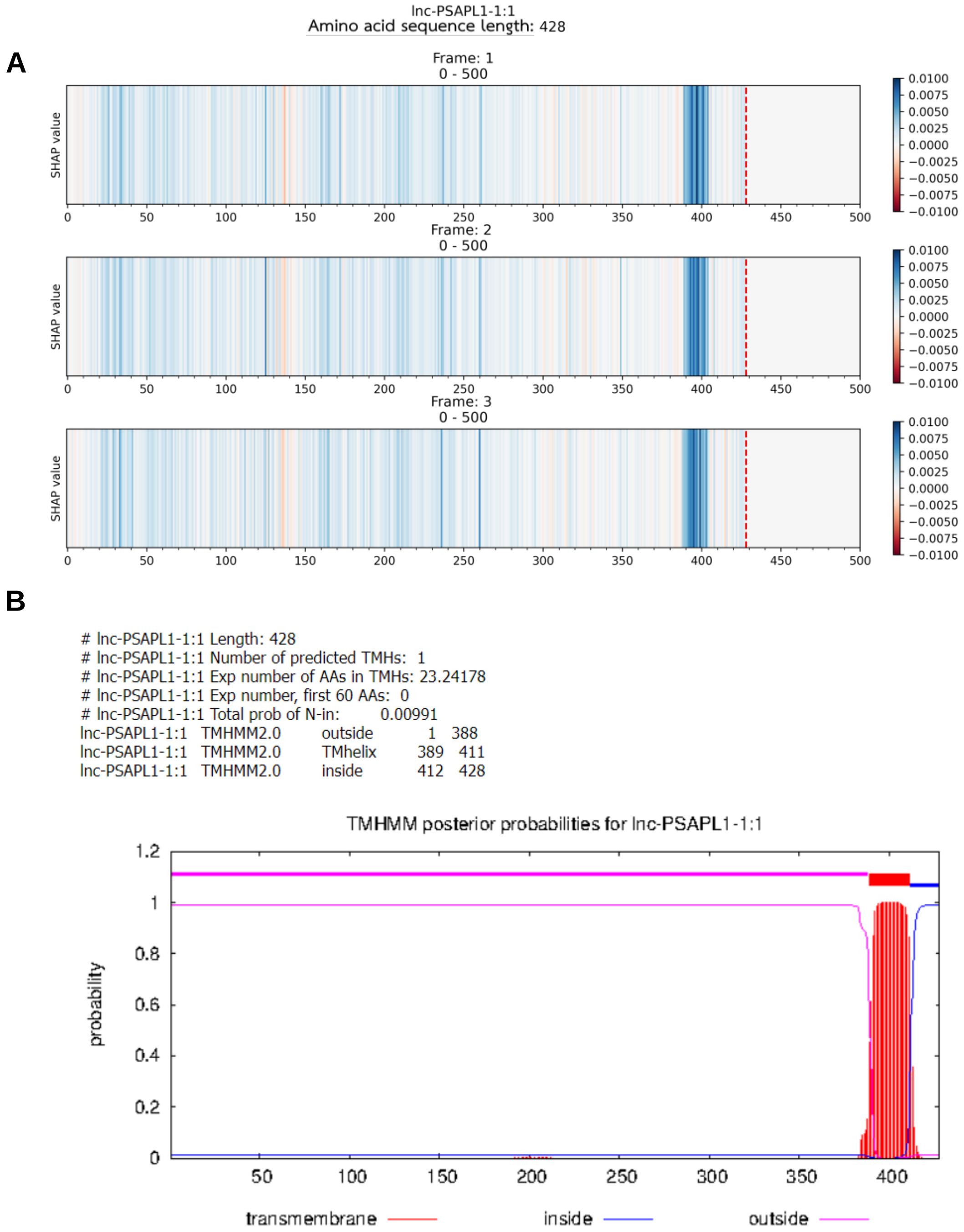

Figure S4. (**A**) The explanation result of Xlnc1DCNN and (**B**) the prediction result of the transmembrane helices by TMHMM program on the true positive sequence, lnc-PSAPL1-1:1.

A

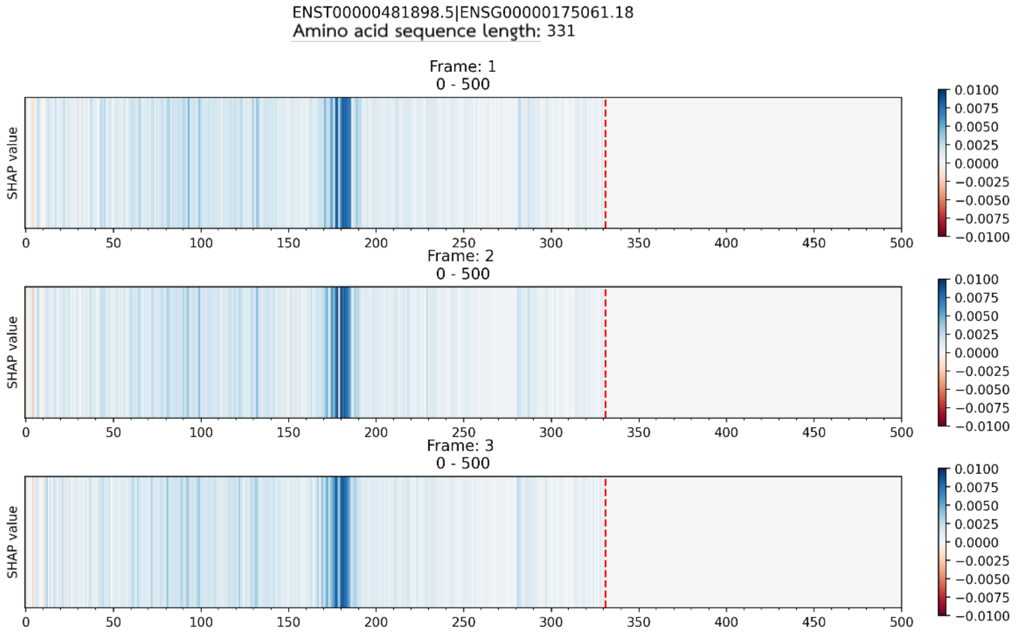

B

```
# ENST00000481898.5 Length: 330
# ENST00000481898.5 Number of predicted TMHs:  1
# ENST00000481898.5 Exp number of AAs in TMHs: 25.62541
# ENST00000481898.5 Exp number, first 60 AAs:  0
# ENST00000481898.5 Total prob of N-in:        0.04000
ENST00000481898.5   TMHMM2.0    outside      1   166
ENST00000481898.5   TMHMM2.0    TMhelix    167   186
ENST00000481898.5   TMHMM2.0    inside     187   330
```

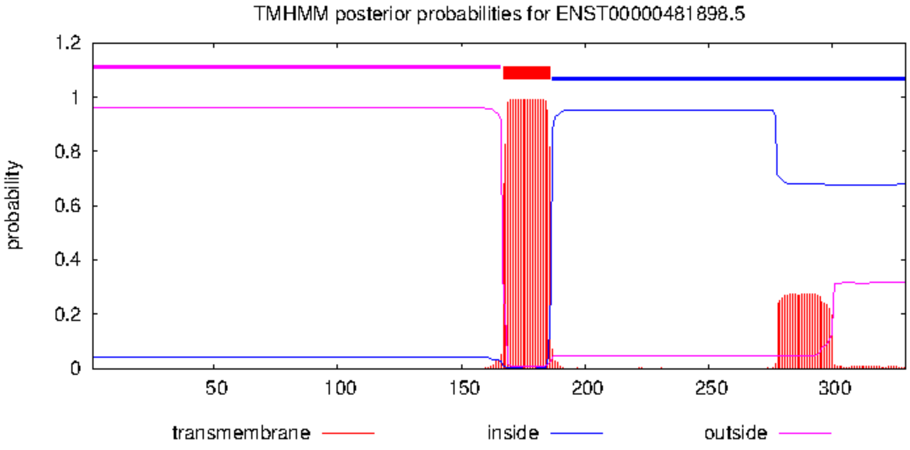

Figure S5. (**A**) The explanation result of Xlnc1DCNN and (**B**) the prediction result of the transmembrane helices by TMHMM program on the true positive sequence, ENST00000481898.5.

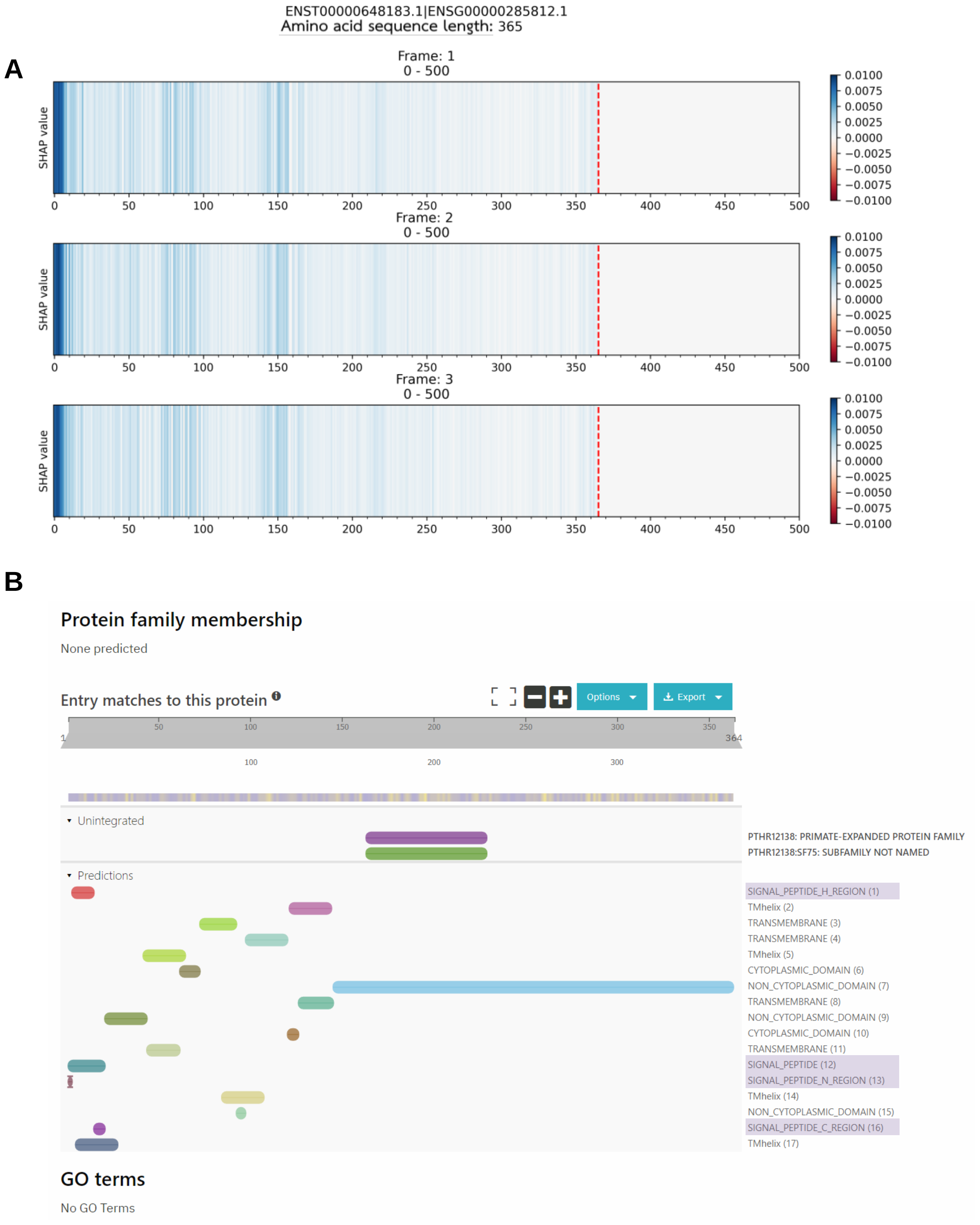

Figure S6. (**A**) The explanation result of Xlnc1DCNN and (**B**) the signal peptide identified by InterPro on the true positive sequence, ENST00000648183.1.

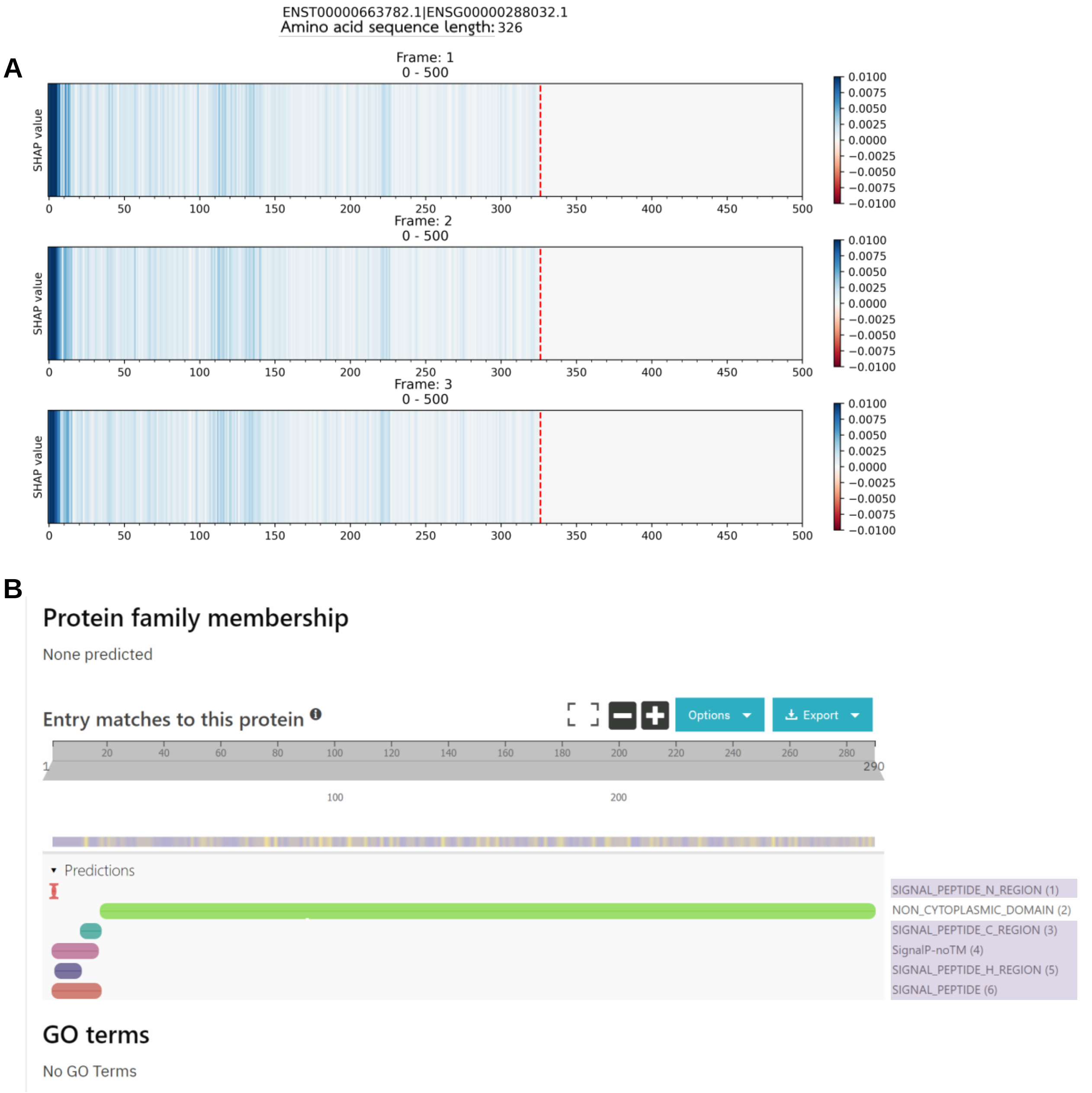

Figure S7. (**A**) The explanation result of Xlnc1DCNN and (**B**) the signal peptide identified by InterPro on the true positive sequence, ENST00000663782.1.

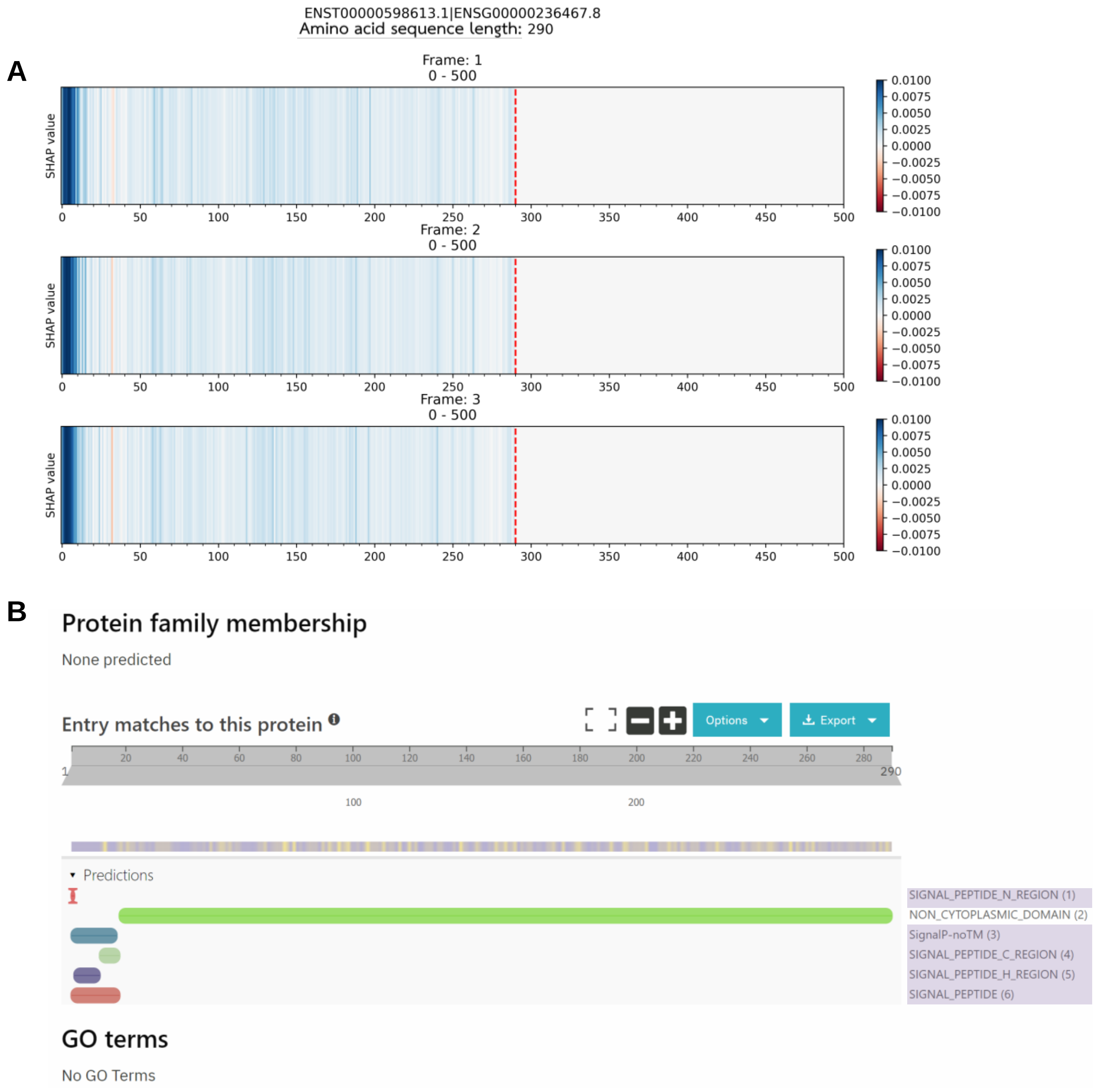

Figure S8. (**A**) The explanation result of Xlnc1DCNN and (**B**) the signal peptide identified by InterPro on the true positive sequence, ENST00000598613.1.

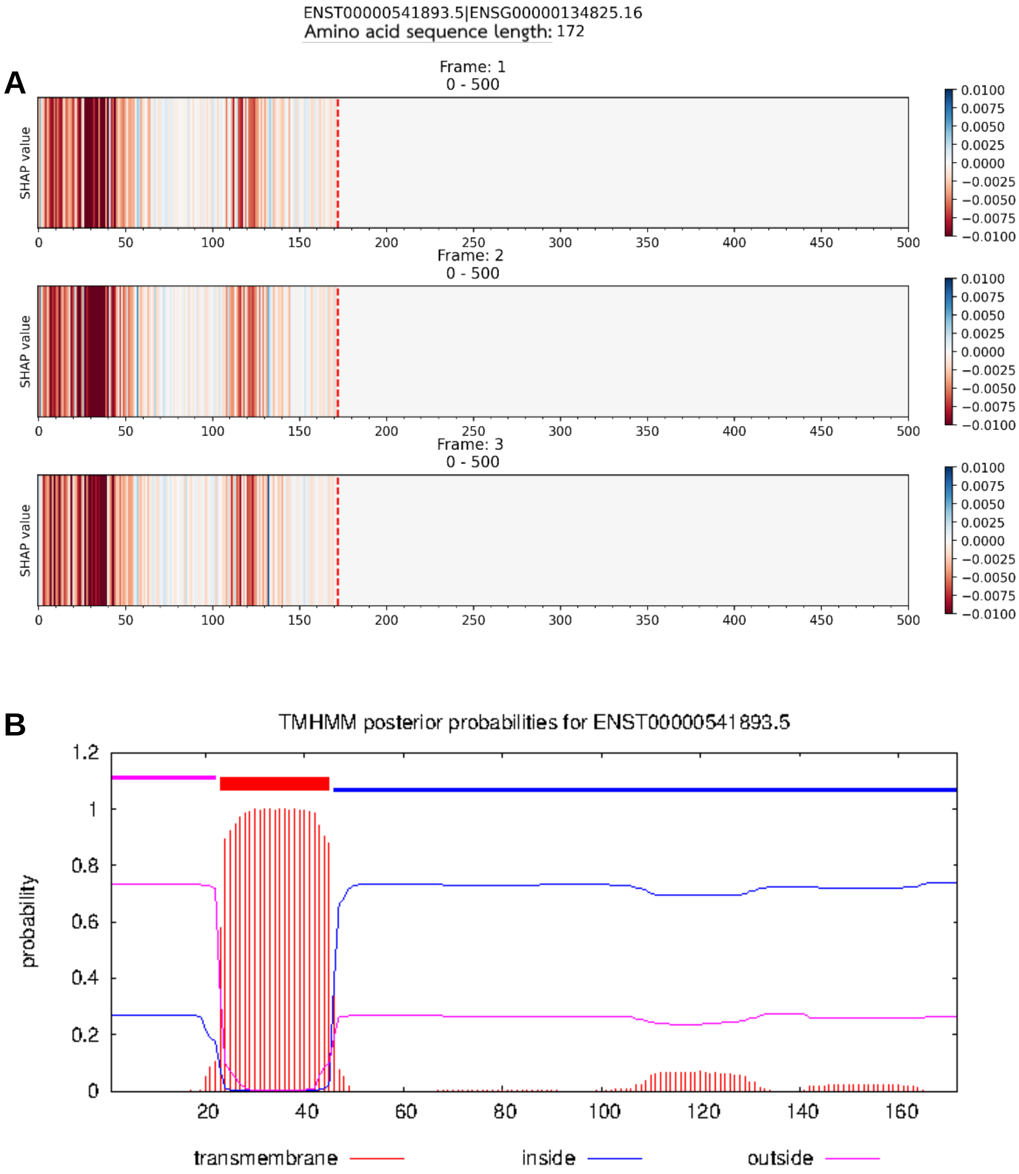

Figure S9. (**A**) The explanation result of Xlnc1DCNN and (**B**) the prediction result of the transmembrane helices by TMHMM program on the true negative sequence, ENST00000541893.5.

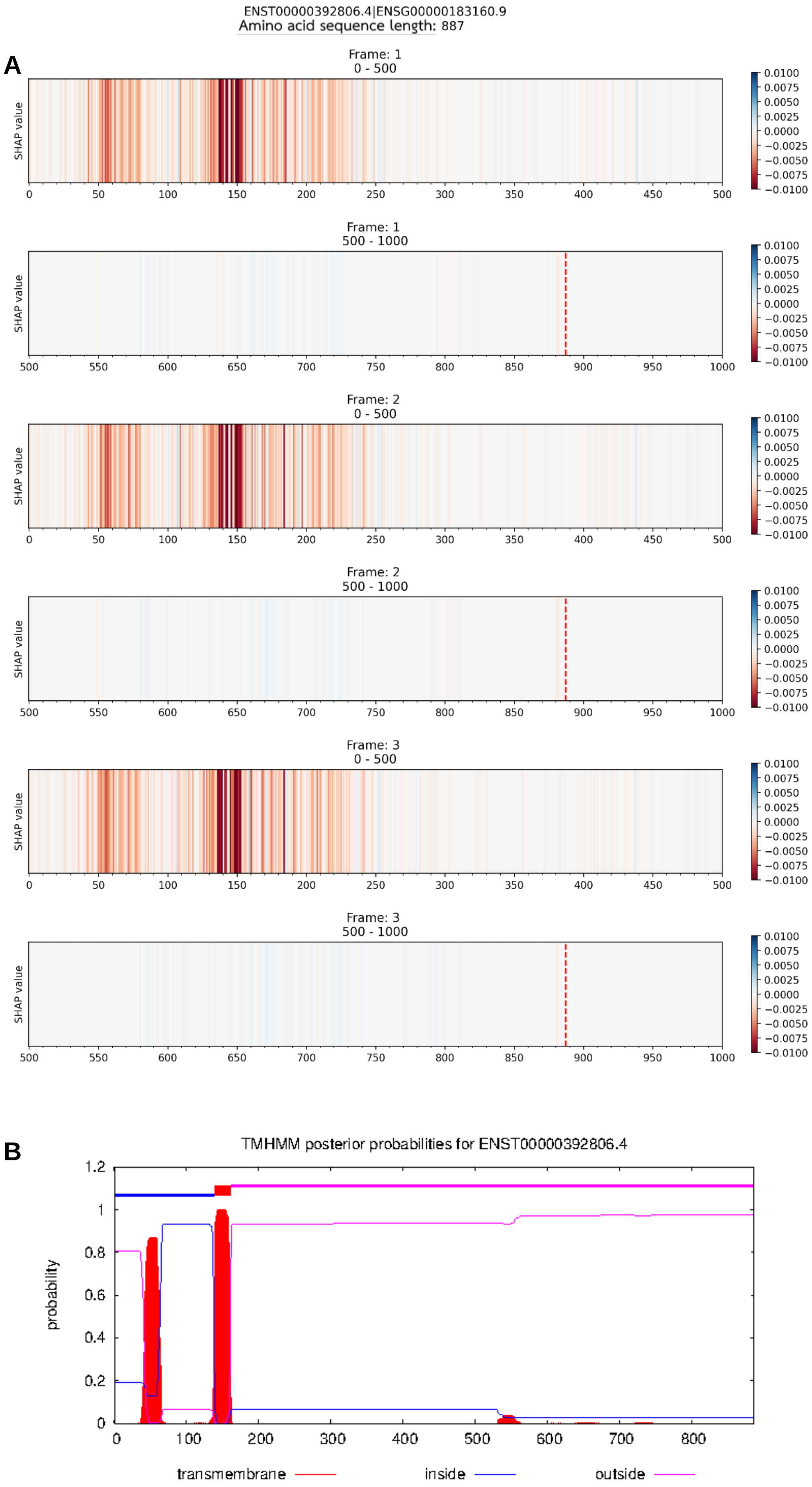

Figure S10. (**A**) The explanation result of Xlnc1DCNN and (**B**) the prediction result of the transmembrane helices by TMHMM program on the true negative sequence, ENST00000392806.4.

A

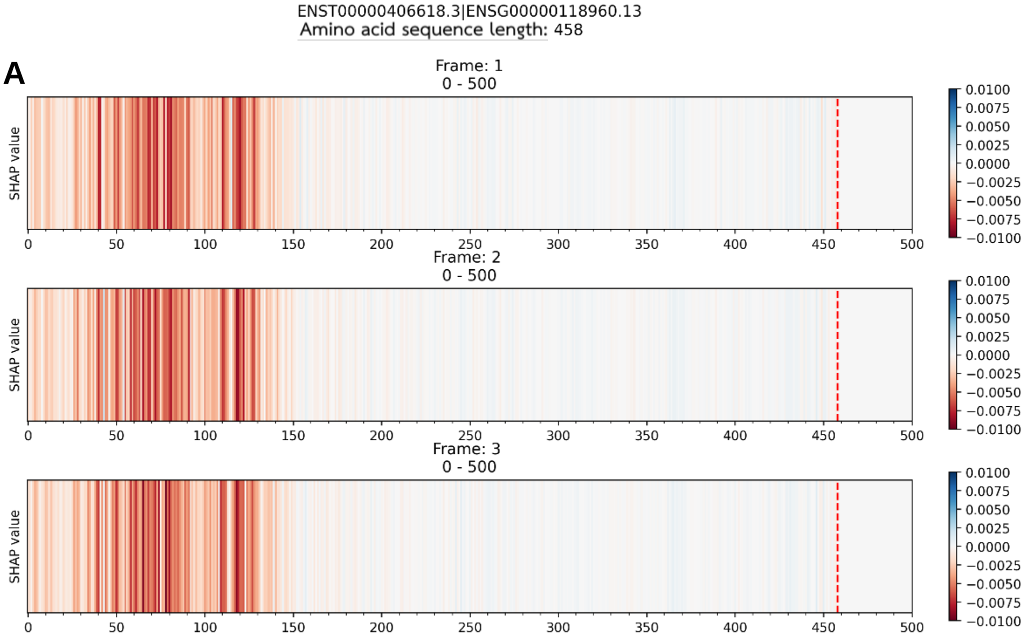

B

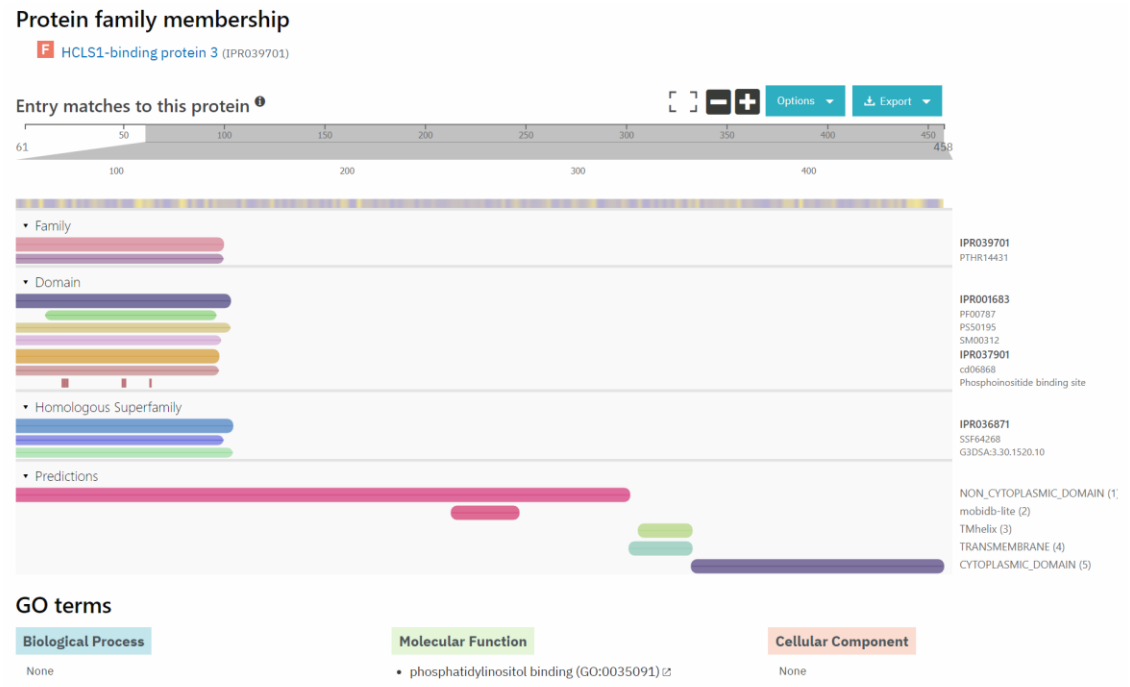

Figure S11. (**A**) The explanation result of Xlnc1DCNN and (**B**) the HCLS1-binding protein 3 (IPR039701) family and Phox homology (IPR001683) domain identified by InterPro on the true negative sequence, ENST00000406618.3.

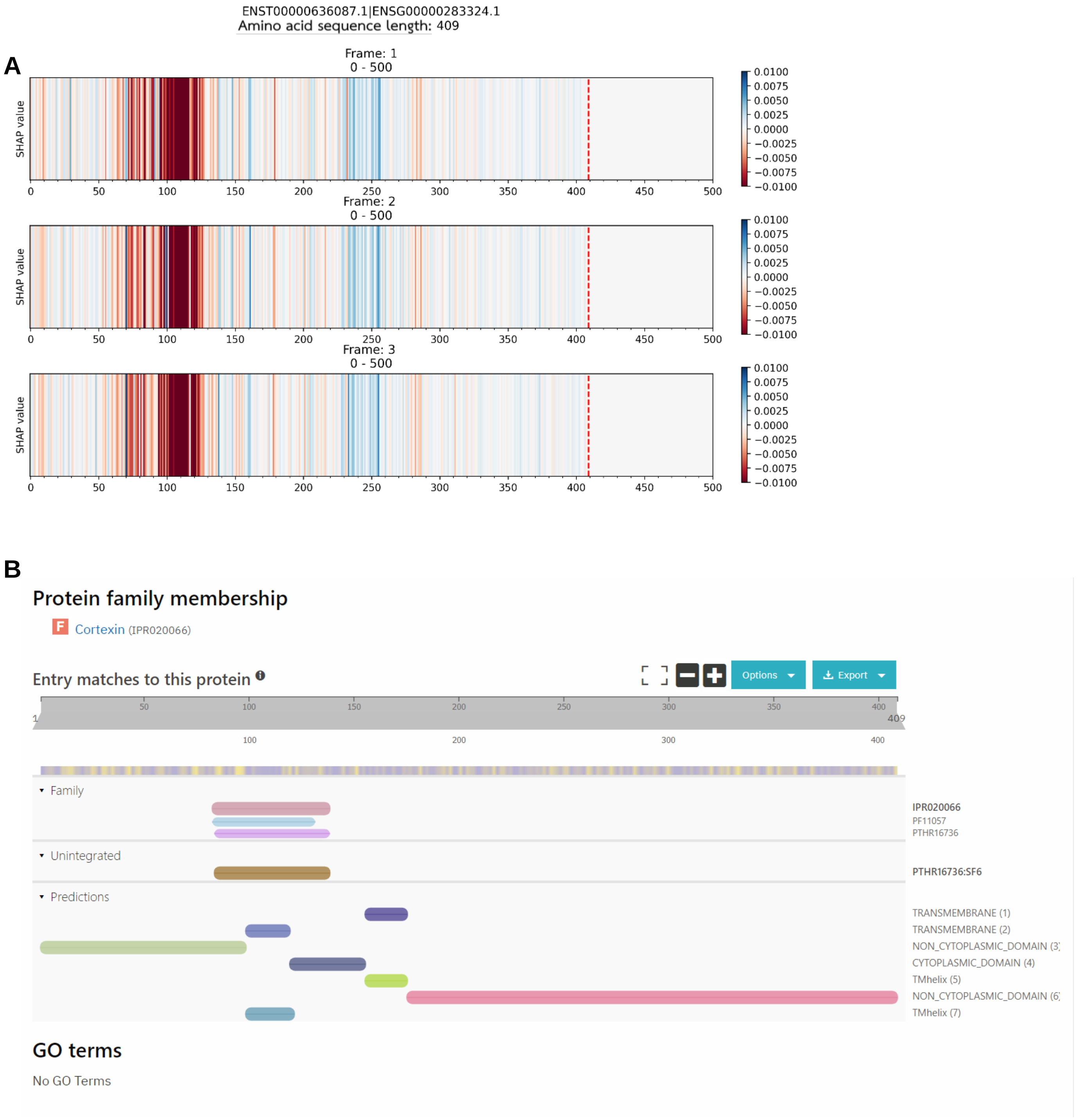

Figure S12. (**A**) The explanation result of Xlnc1DCNN and (**B**) the Cortexin (IPR020066) family identified by InterPro on the true negative sequence, ENST00000636087.1.

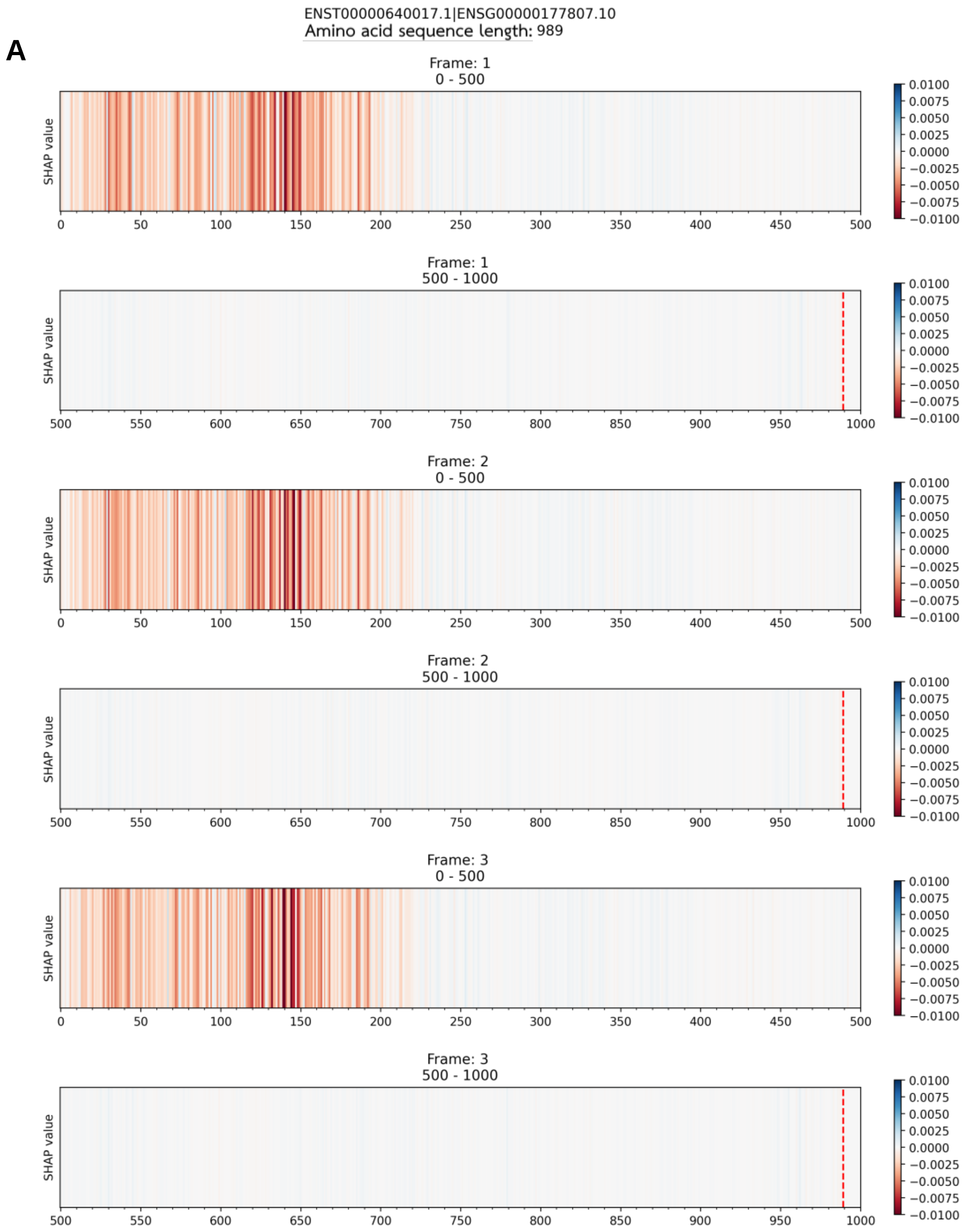

ENST00000640017.1|ENSG00000177807.10
Amino acid sequence length: 989
A
Frame: 1
0 - 500
Frame: 1
500 - 1000
Frame: 2
0 - 500
Frame: 2
500 - 1000
Frame: 3
0 - 500
Frame: 3
500 - 1000
SHAP value
0.0100
0.0075
0.0050
0.0025
0.0000
−0.0025
−0.0050
−0.0075
−0.0100

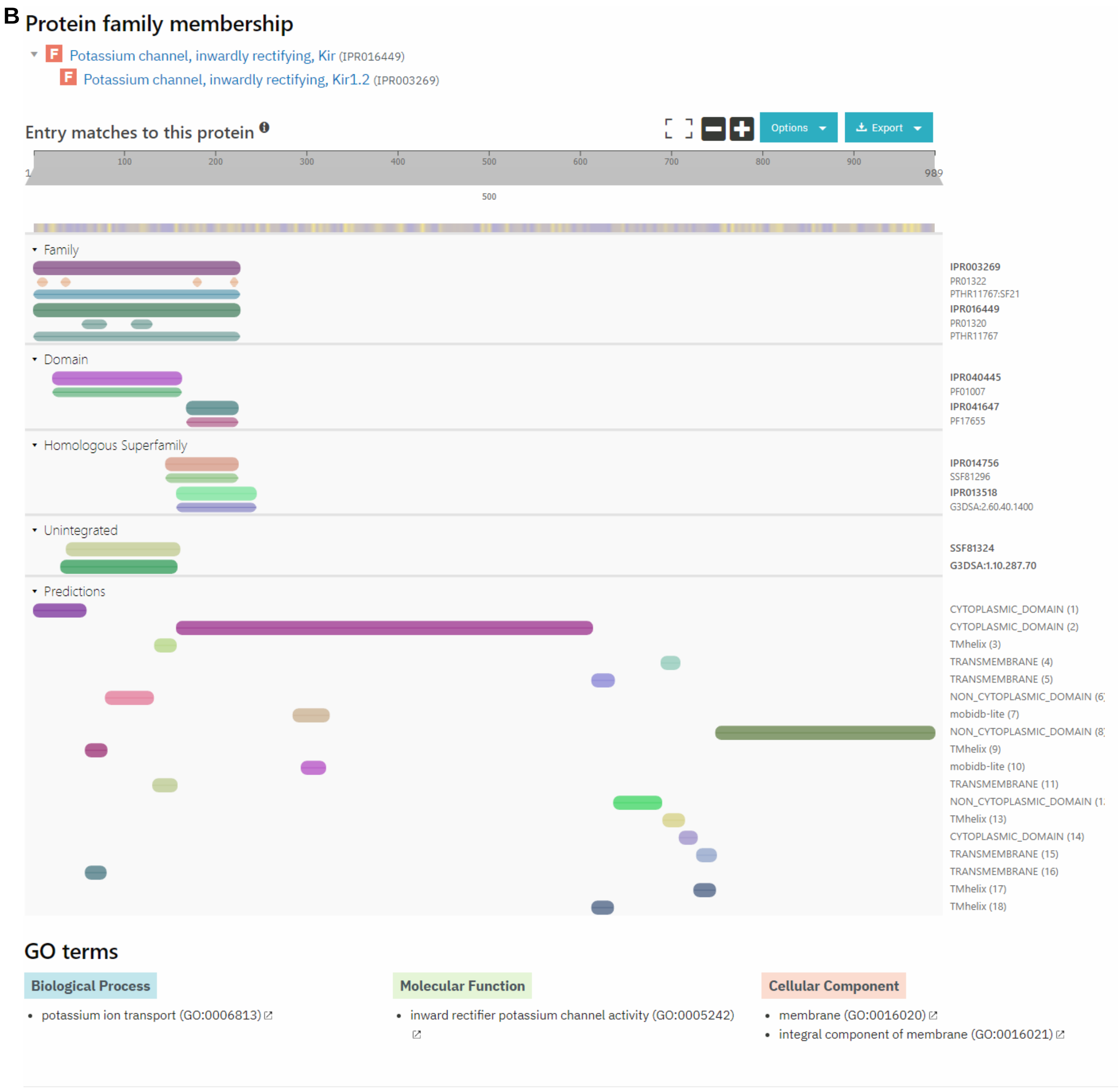

Figure S13. (**A**) The explanation result of Xlnc1DCNN and (**B**) the Potassium channel, inwardly rectifying, Kir (IPR016449), Potassium channel, inwardly rectifying, Kir1.2 (IPR003269) families and Potassium channel, inwardly rectifying, transmembrane domain (IPR040445), Inward rectifier potassium channel, C-terminal (IPR041647) domains identified by InterPro on the true negative sequence, ENST00000640017.1.

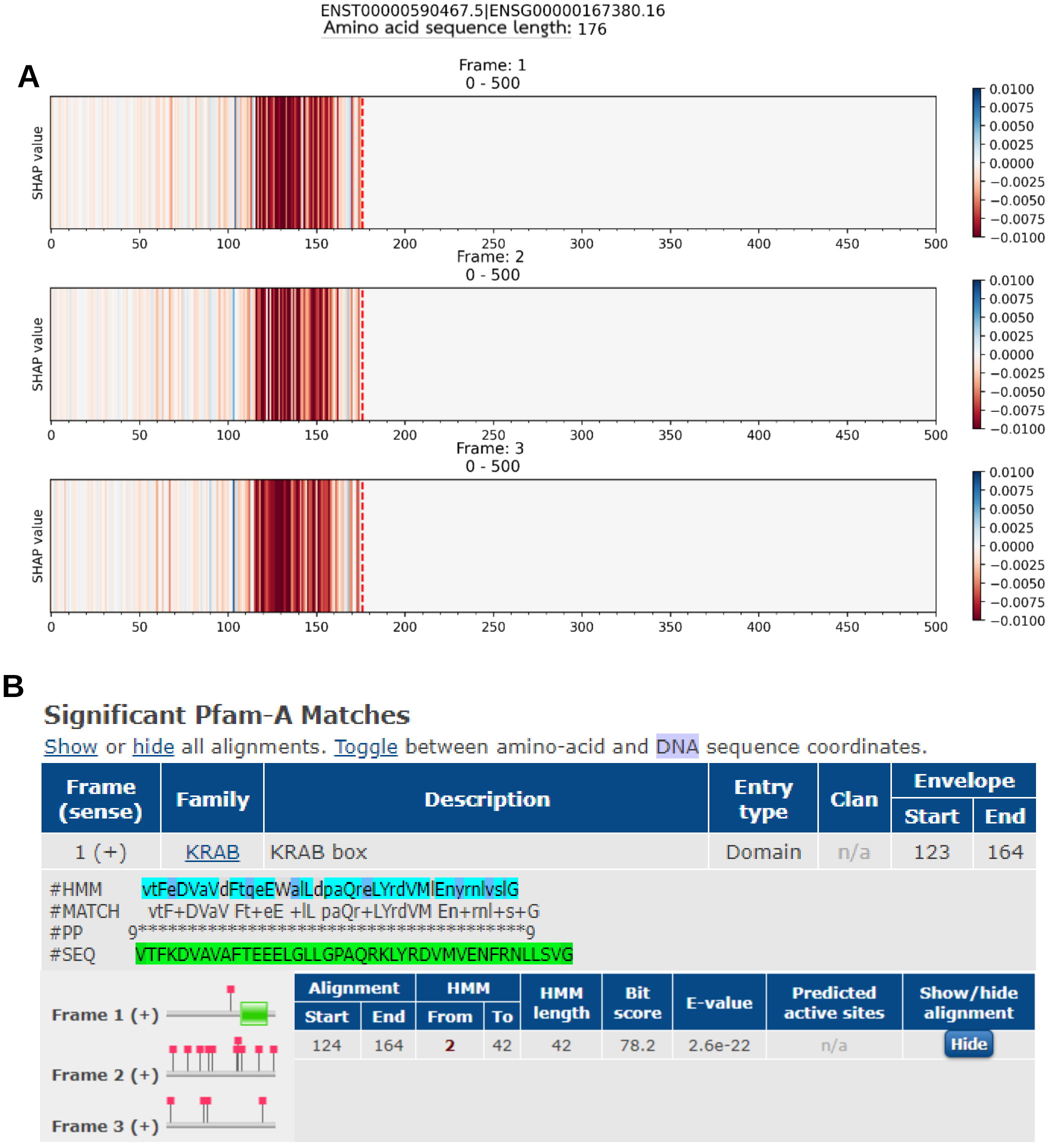

Figure S14. (**A**) The explanation result of Xlnc1DCNN and (**B**) the KRAB domain identified by Pfam on the true negative sequence, ENST00000590467.5.

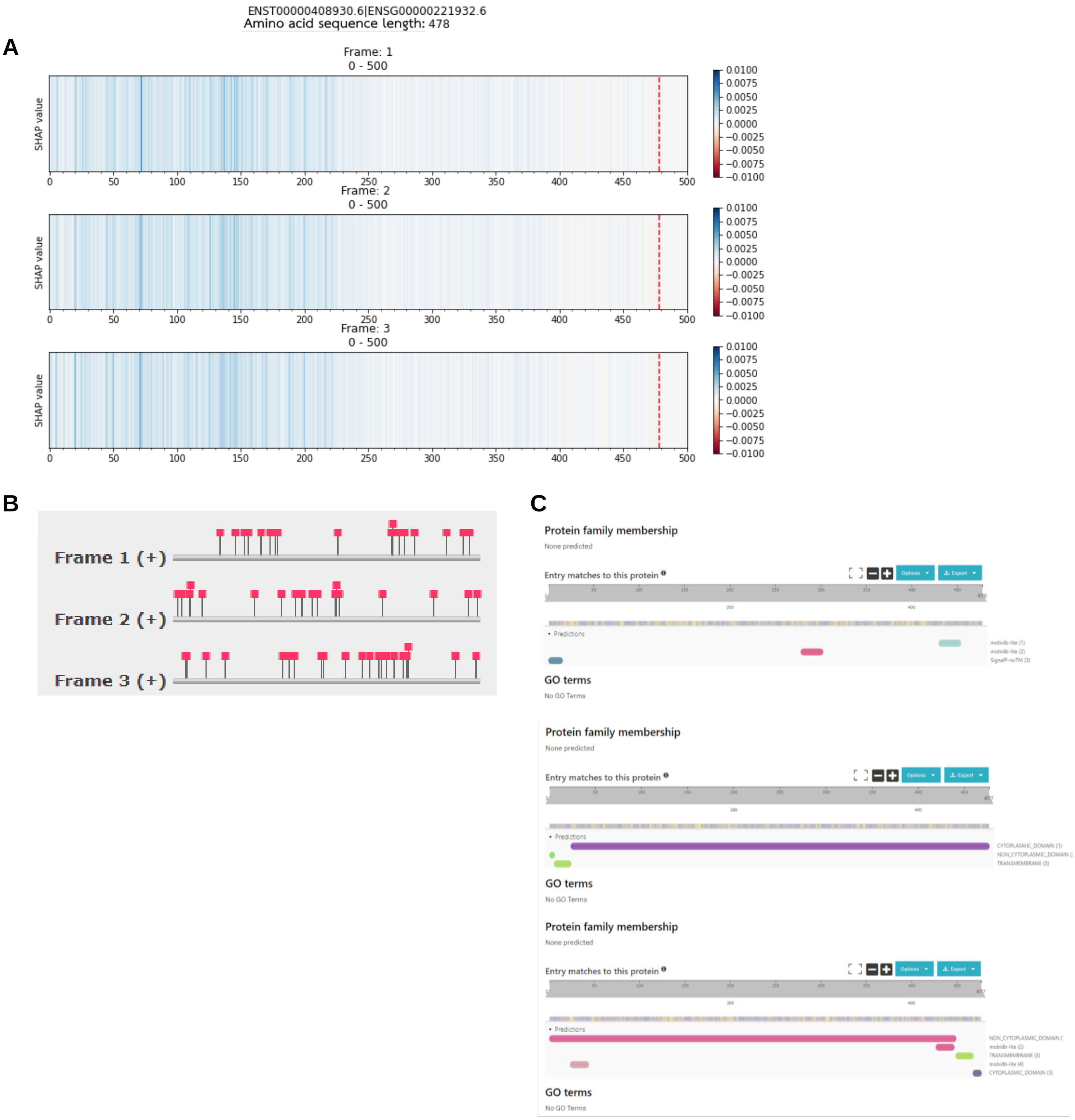

Figure S15. (**A**) The explanation result of Xlnc1DCNN, (**B**) The identification result from Pfam, and (**C**) The identification results from InterPro on the false positive sequence, ENST00000408930.6. Pfam and InterPro could not identify any protein domains or families within the sequence.

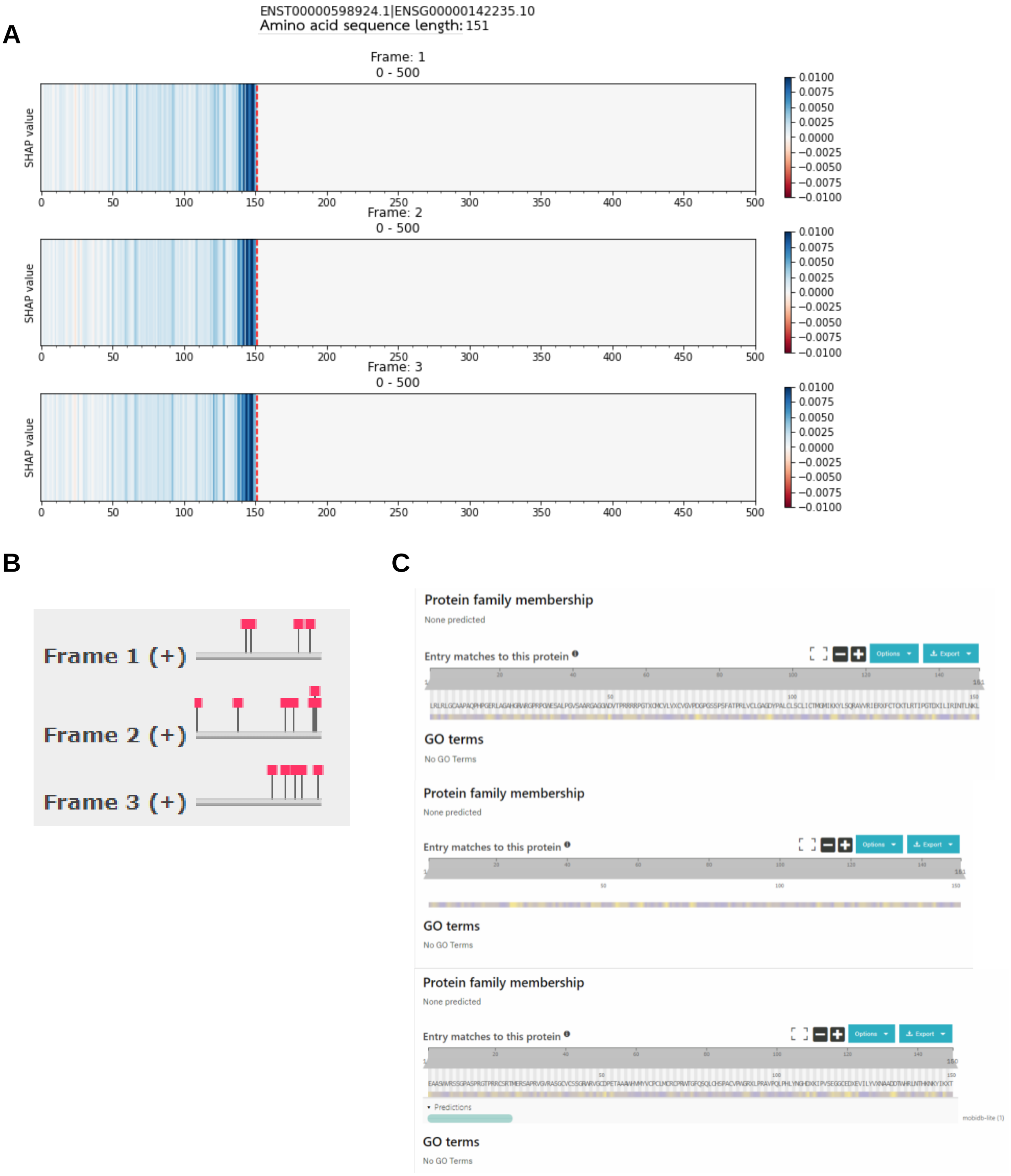

Figure S16. (**A**) The explanation result of Xlnc1DCNN, (**B**) The identification result from Pfam, and (**C**) The identification results from InterPro on the false positive sequence, ENST00000598924.1. Pfam and InterPro could not identify any protein domains or families within the sequence.

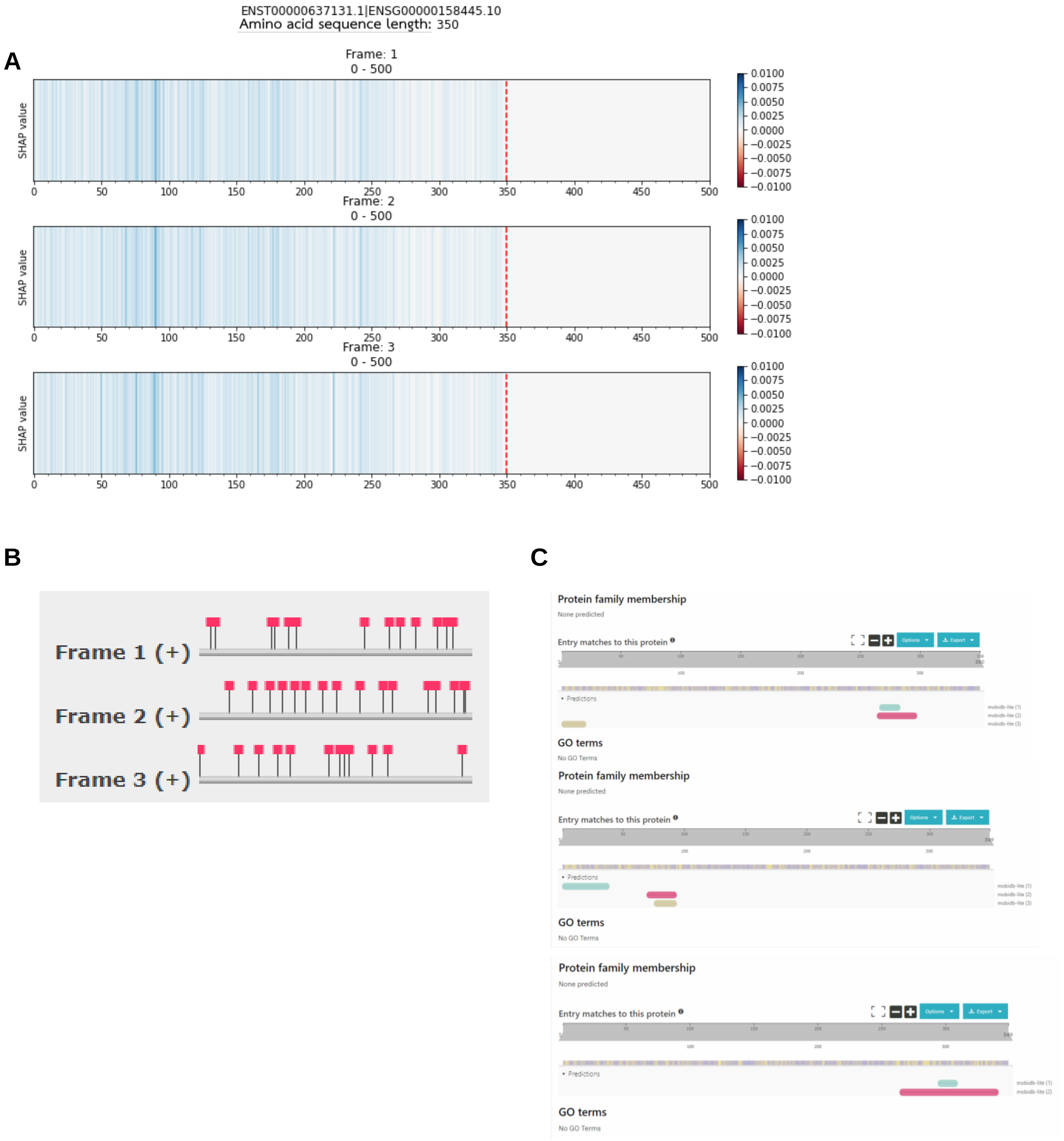

Figure S17. (**A**) The explanation result of Xlnc1DCNN, (**B**) The identification result from Pfam, and (**C**) The identification results from InterPro on the false positive sequence, ENST00000637131.1. Pfam and InterPro could not identify any protein domains or families within the sequence.

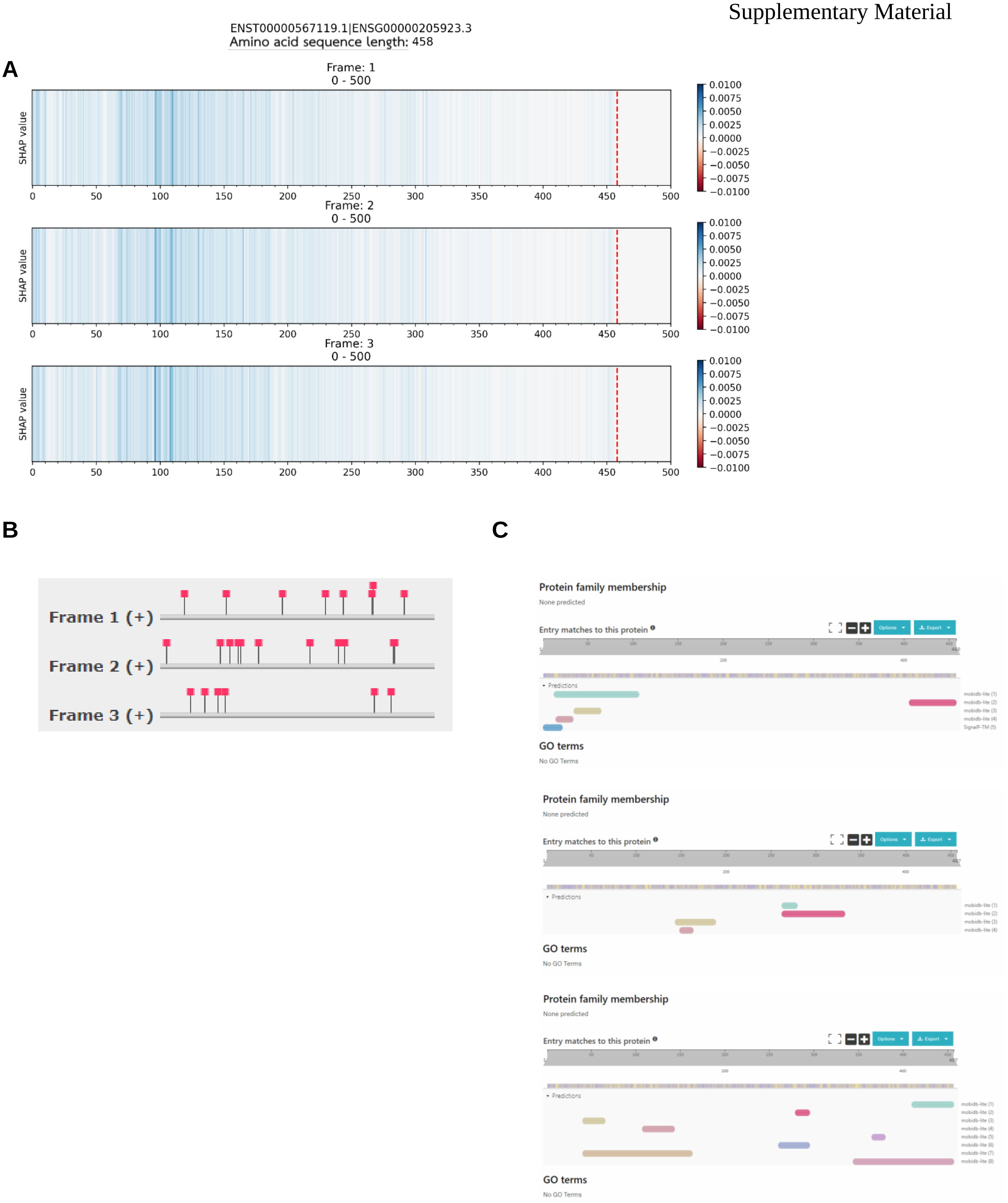

Figure S18. (**A**) The explanation result of Xlnc1DCNN, (**B**) The identification result from Pfam, and (**C**) The identification results from InterPro on the false positive sequence, ENST00000567119.1. Pfam and Interpro could not identify any protein domains or families within the sequence.

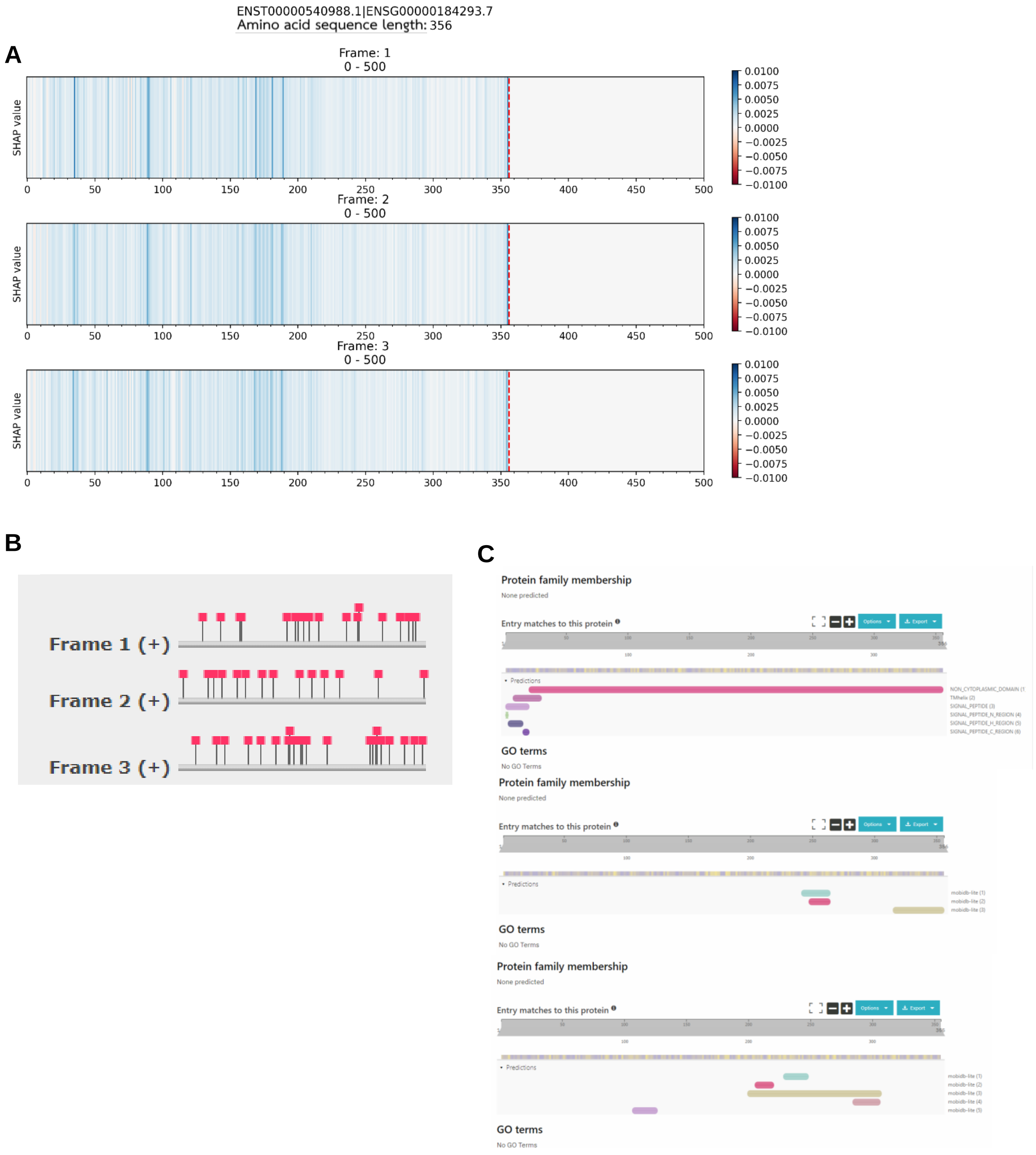

Figure S19. (**A**) The explanation result of Xlnc1DCNN, (**B**) The identification result from Pfam, and (**C**) The identification results from InterPro on the false positive sequence, ENST00000540988.1. Pfam and InterPro could not identify any protein domains or families within the sequence.

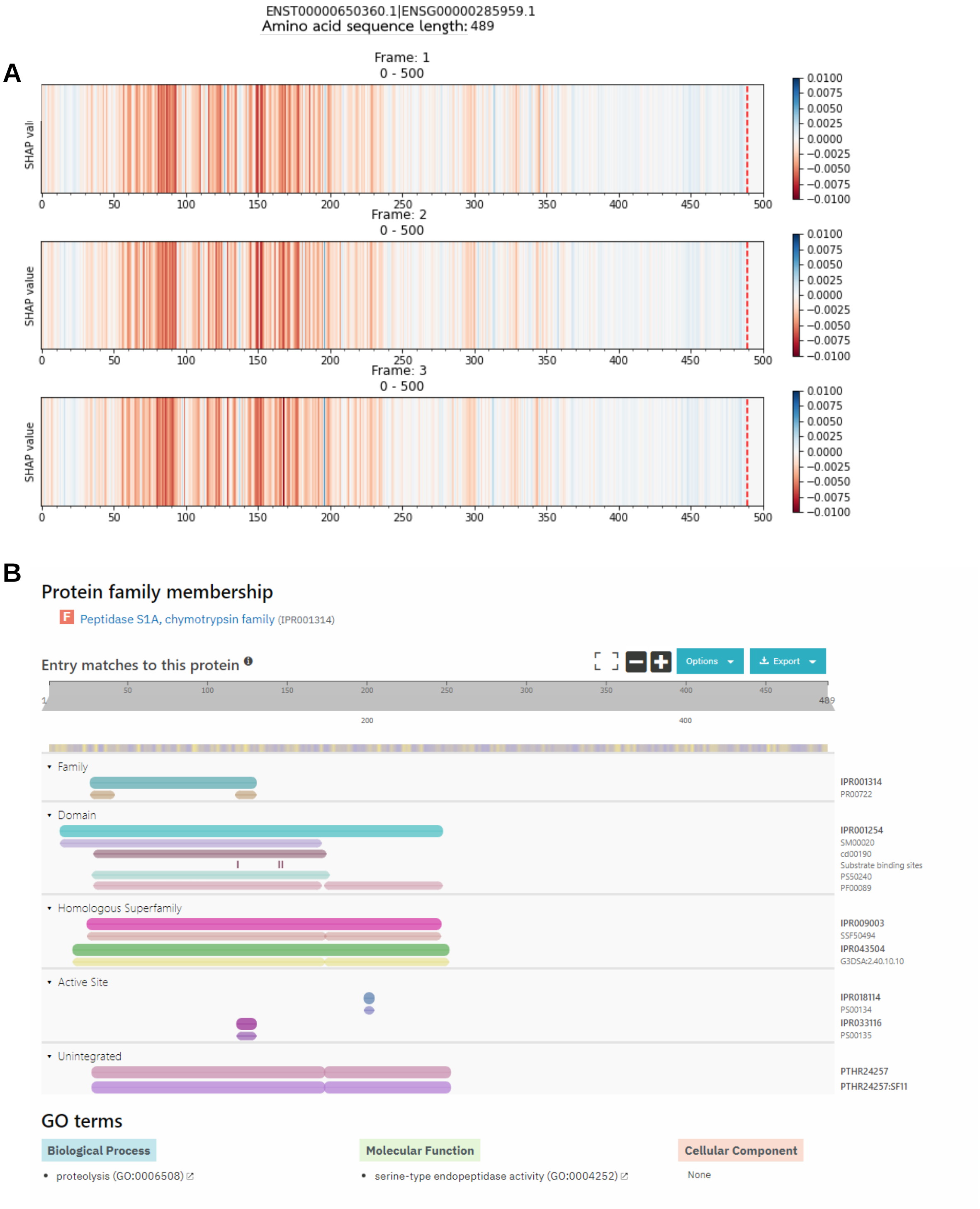

Figure S20. (**A**) The explanation result of Xlnc1DCNN and (**B**) the Peptidase S1A, chymotrypsin family (IPR001314) and Serine proteases, trypsin domain (IPR001254) identified by InterPro on the false negative sequence, ENST00000650360.1.

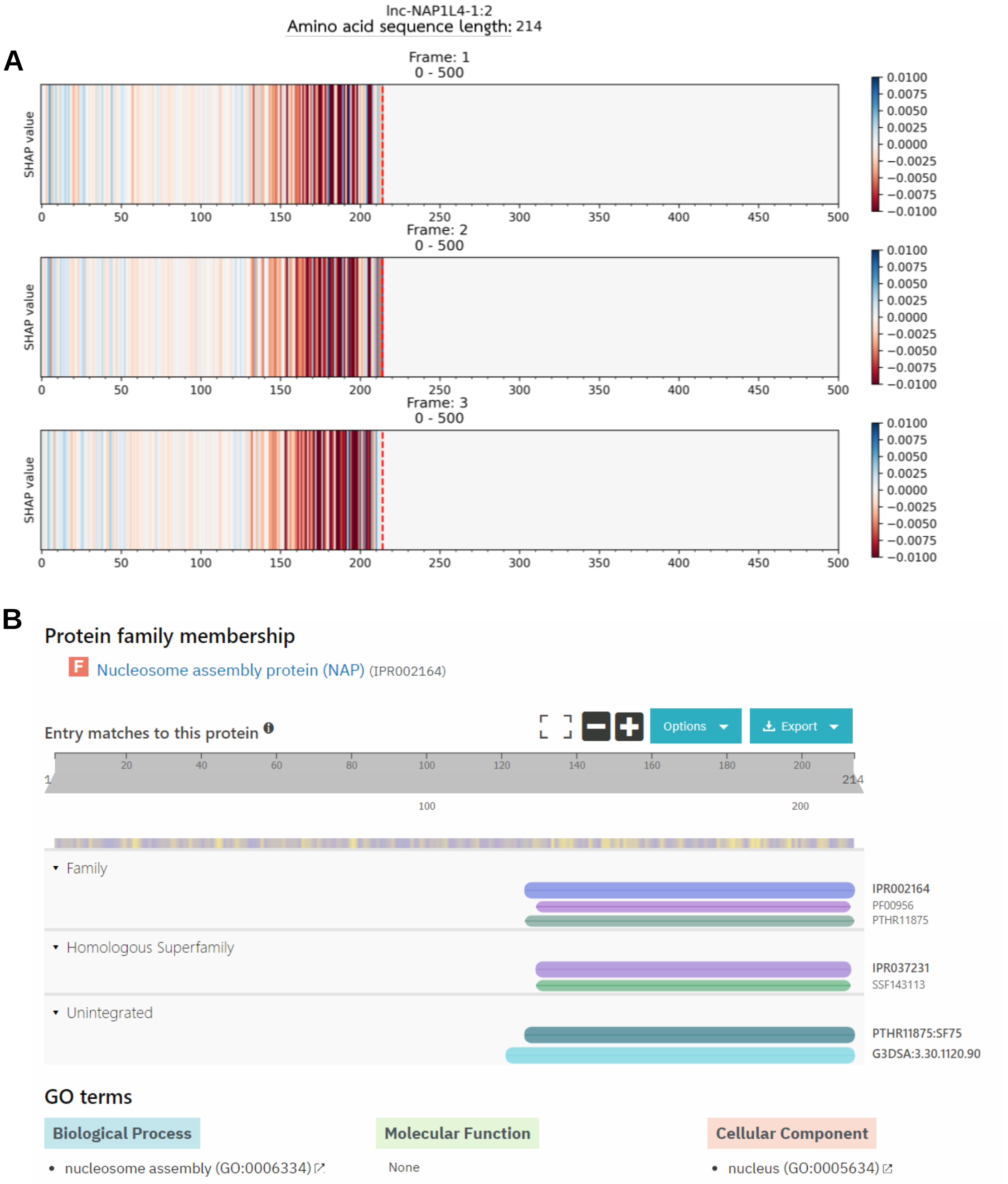

Figure S21. (**A**) The explanation result of Xlnc1DCNN and (**B**) the Nucleosome assembly protein (NAP) (IPR002164) family identified by InterPro on the false negative sequence, lnc-NAP1L4-1:2.

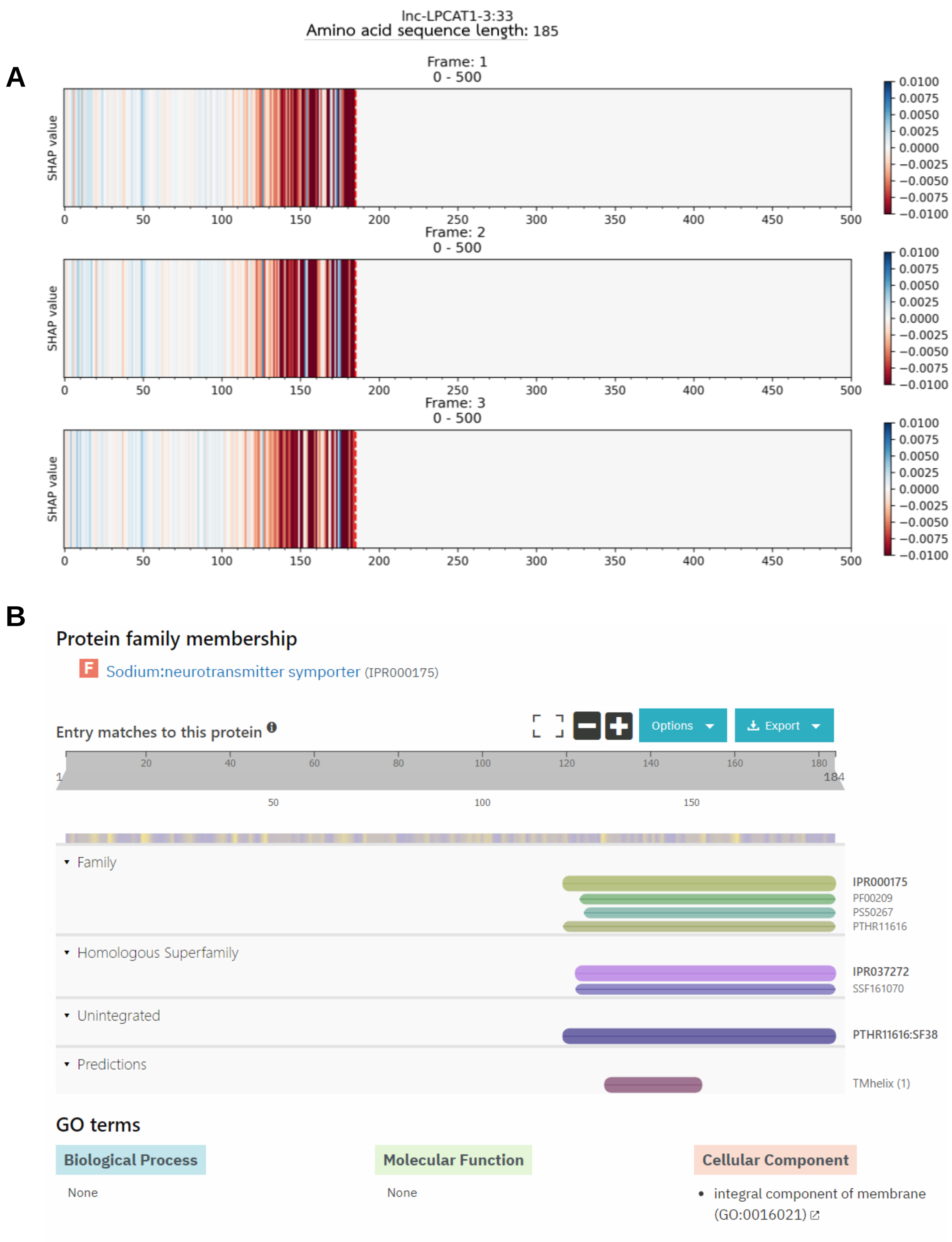

Figure S22. (**A**) The explanation result of Xlnc1DCNN and (**B**) the Sodium:neurotransmitter symporter (IPR000175) family identified by InterPro on the false negative sequence, lnc-LPCAT1-3:33.

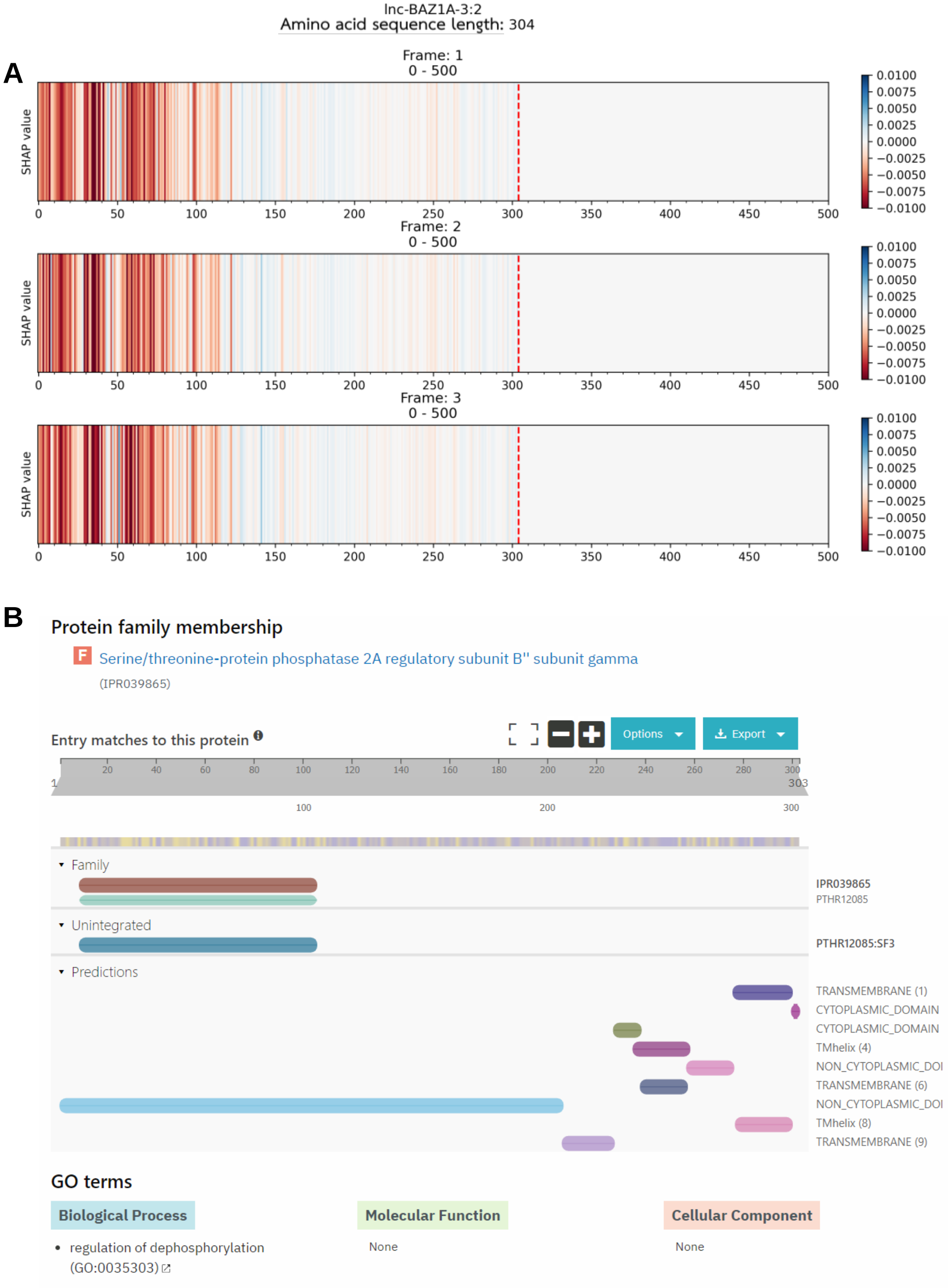

Figure S23. (**A**) The explanation result of Xlnc1DCNN and (**B**) the Serine/threonine-protein phosphatase 2A regulatory subunit B'' subunit gamma (IPR039865) family identified by InterPro on the false negative sequence, lnc-BAZ1A-3:2.

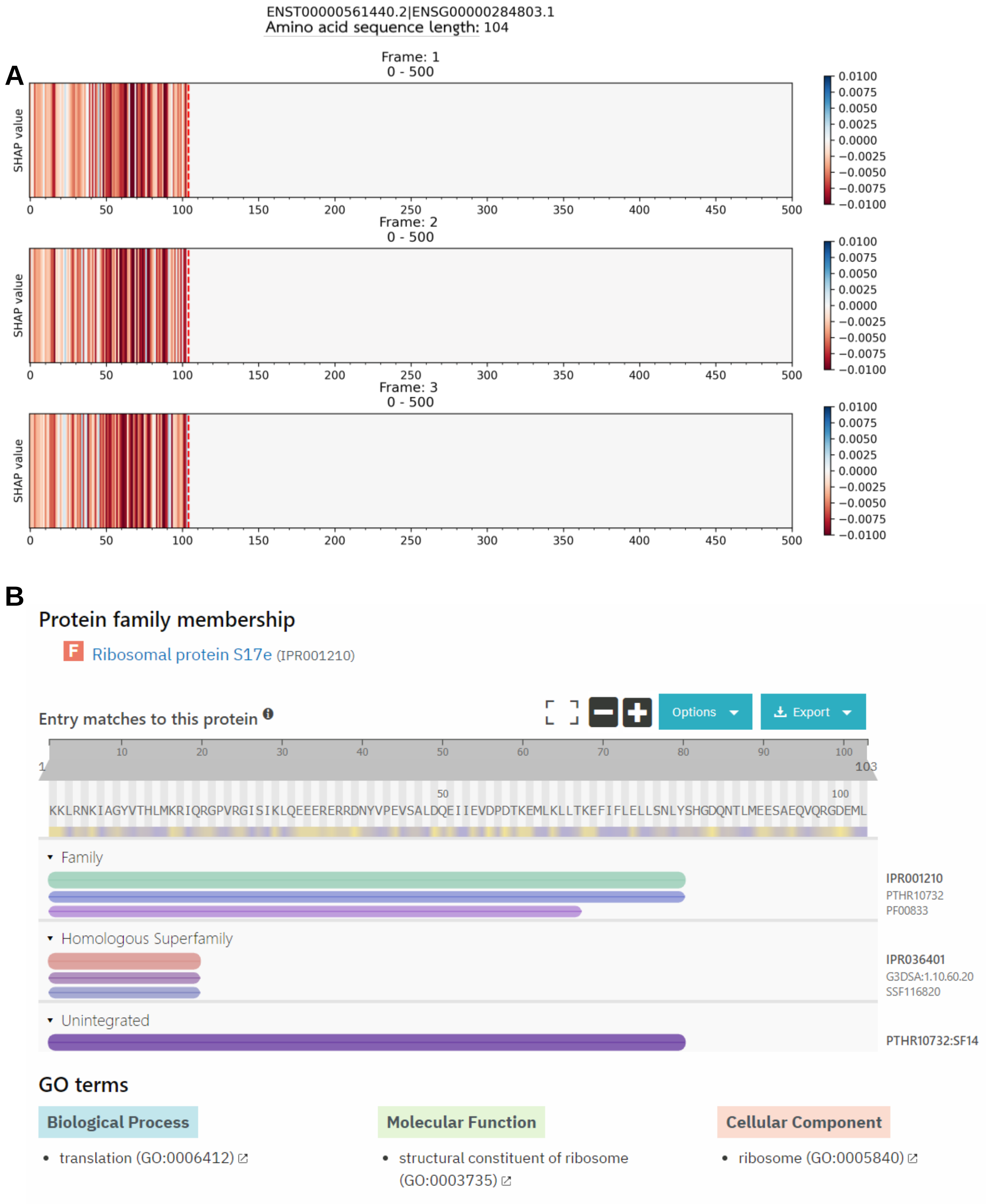

Figure S24. (**A**) The explanation result of Xlnc1DCNN and (**B**) the Ribosomal protein S17e (IPR001210) family identified by InterPro on the false negative sequence, ENST00000561440.2.

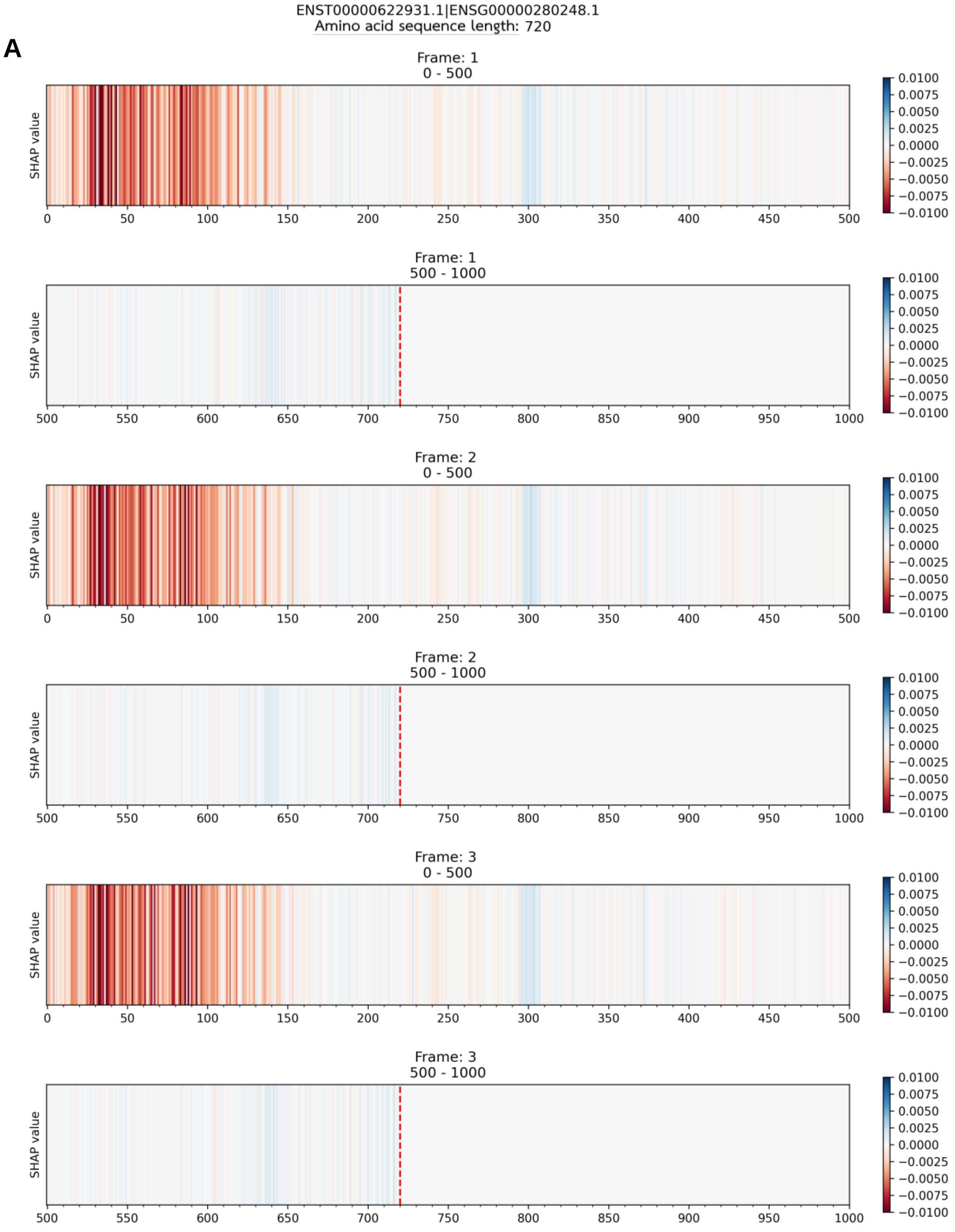
ENST00000622931.1|ENSG00000280248.1
Amino acid sequence length: 720
A
Frame: 1
0 - 500
SHAP value
0 50 100 150 200 250 300 350 400 450 500
0.0100 0.0075 0.0050 0.0025 0.0000 −0.0025 −0.0050 −0.0075 −0.0100
Frame: 1
500 - 1000
SHAP value
500 550 600 650 700 750 800 850 900 950 1000
Frame: 2
0 - 500
Frame: 2
500 - 1000
Frame: 3
0 - 500
Frame: 3
500 - 1000

B

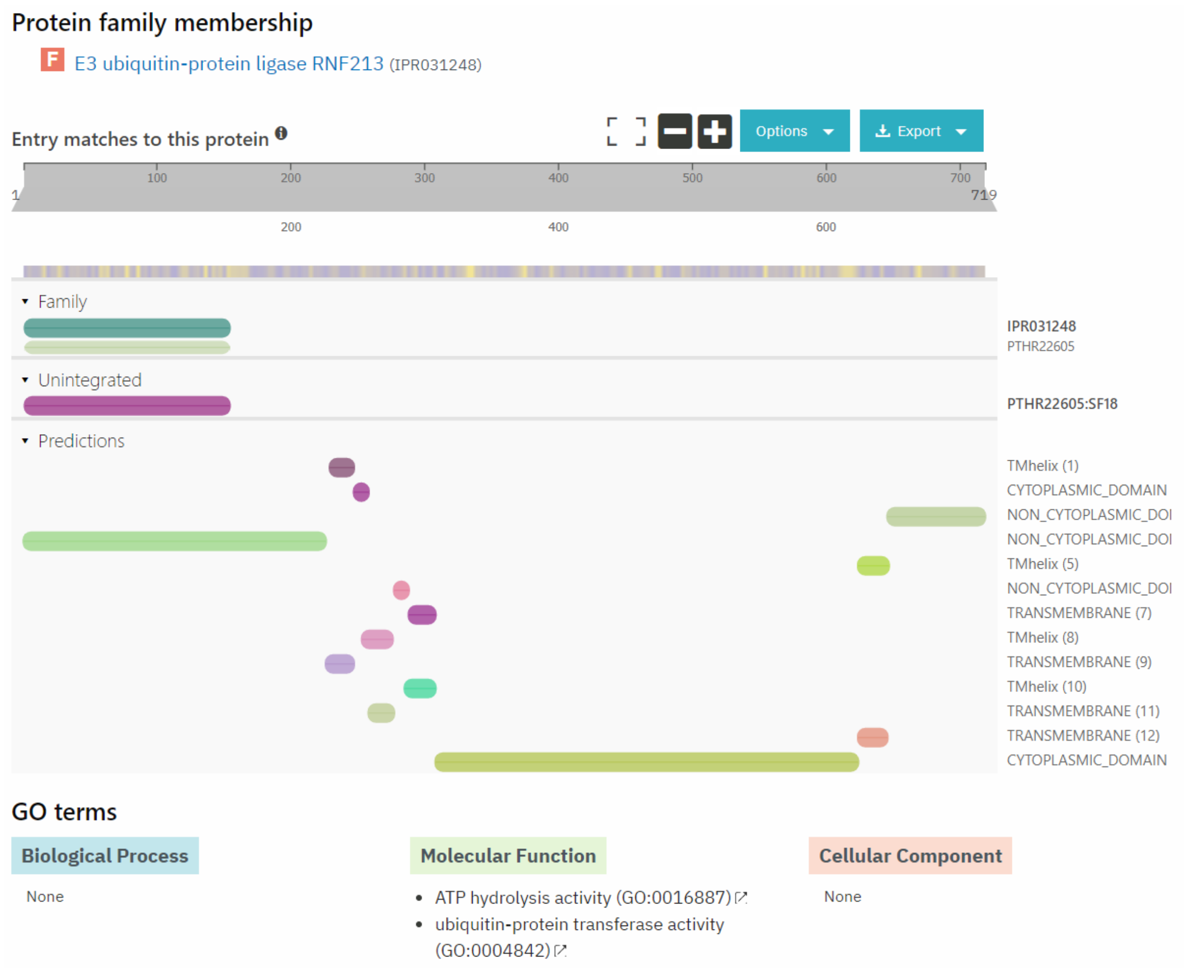

Figure S25. (**A**) The explanation result of Xlnc1DCNN and (**B**) the E3 ubiquitin-protein ligase RNF213 (IPR031248) family identified by InterPro on the false negative sequence, ENST00000622931.1.
